# Climate warming promotes growth in Himalayan alpine cushion plants but threatens survival through increased extreme snowfall

**DOI:** 10.1111/nph.70206

**Published:** 2025-05-12

**Authors:** Veronika Jandova, Jan Altman, Hana Sehadova, Martin Macek, Pavel Fibich, Adam Taylor Ruka, Jiri Dolezal

**Affiliations:** ^1^ Institute of Botany of the Czech Academy of Science Zámek 1 CZ 252 43 Průhonice Czech Republic; ^2^ Faculty of Science University of South Bohemia Branišovská 1760 CZ 370 05 České Budějovice Czech Republic; ^3^ Laboratory of Microscopy and Histology, Biology Centre Institute of Entomology of the Czech Academy of Sciences Branišovská 1160 CZ 370 05 České Budějovice Czech Republic

**Keywords:** alpine ecosystems, climate warming, frost‐free days, growth–longevity trade‐off, growth ring analyses, herb‐chronology, plant recruitment and growth, subnival plants

## Abstract

- Climate warming stimulates growth and reproduction in cold‐adapted plants but also leads to extreme weather events that may hinder their performance. We examined these predictions in the cold‐arid Himalayan subnival zone at 5900 m, where unprecedented warming and extreme snowfalls occurred over the past three decades. We collected 205 individuals of *Ladakiella klimesii*, analyzing climate influences on their growth and recruitment through annual growth rings.
- Radial growth was highly sensitive to summer temperatures, with warmer conditions significantly enhancing growth. However, increased winter precipitation negatively impacted growth and recruitment by shortening the growing season. Warmer winters and springs, combined with autumn snow cover, favored recruitment, while extreme late winter and summer snowfall disrupted growth and recruitment through intensified soil disturbances.
- We also found a trade‐off between growth rate and longevity: Plants established during warmer periods grow rapidly but have shorter lifespans, whereas those emerging in colder conditions grow more slowly yet persist longer, with implications for long‐term population stability.
- These findings highlight the complex relationship between growth, longevity, and survival in a shifting climate. Although warming promotes growth, it may also decrease longevity and population persistence. The rising frequency of extreme snowfall presents new survival challenges for the world's highest‐occurring plants.

## Introduction

Understanding the impact of climate change, particularly current warming trends, on the recruitment and growth of alpine plants is essential for predicting shifts in alpine ecosystems (Dee & Stambaugh, 2019; Centenaro *et al*., 2023; Thakur *et al*., 2024). Although the alpine region has experienced significant warming over the past three decades (Pepin *et al*., 2022), our knowledge of its long‐term effects on alpine plants is limited (Dee & Stambaugh, 2019). While growth responses to climate change are well documented in alpine and arctic shrubs (Rixen *et al*., 2010; Büntgen *et al*., 2015; Myers‐Smith & Hik, 2018; Francon *et al*., 2020), evaluations of long‐term growth and regeneration in alpine forbs, which dominate high‐elevation flora, are scarce (Franklin, 2013; Dolezal *et al*., 2020). Additionally, there is little understanding of the growth dynamics of alpine cushion plants at extreme elevations, such as those reaching 6000 m in the Himalayas (Dolezal *et al*., 2016; Dentant, 2018). Investigating these growth responses to accelerated warming is crucial for predicting future impacts on high‐elevation ecosystems (Ferrarini *et al*., 2019; Leng *et al*., 2023).

Cold temperatures significantly constrain growth in alpine environments, but recent evidence suggests that accelerated warming and altered precipitation patterns may further hinder the growth of some cold‐adapted alpine plants due to increased droughts and extreme weather events (Dolezal *et al*., 2020; Bonanomi *et al*., 2023; Rai *et al*., 2024). Alpine plants, particularly rosette and cushion species, have adapted to cold through compact canopies that provide frost insulation and nutrient‐rich litter (Dvorský *et al*., 2013; Aubert *et al*., 2014; Chondol *et al*., 2024). These adaptations enhance thermal conditions, soil moisture, and nutrient availability, promoting resilience compared to larger shrubs and trees (Callaway, 2007; Molenda *et al*., 2012). However, their ability to trap heat may become detrimental with rising temperatures (Gauslaa, 1984), potentially leading to growth limitations and dieback, as seen during recent heatwaves (Duchicela *et al*., 2021; Bonanomi *et al*., 2023).

Climate warming is also expected to alter plant growth trajectories by changing the balance between growth rate, lifespan, and resource allocation (Körner, 2017; Büntgen *et al*., 2019). Increased temperatures generally enhance metabolic activity and photosynthesis in high elevation, leading to faster early growth and earlier peak growth rates (Dolezal *et al*., 2019). Warming may shorten developmental phases, causing earlier maturity and limiting the development of durable tissues, constraining maximum lifespan (Bigler & Veblen, 2009; Chondol *et al*., 2023). The rapid growth can result in higher respiration costs, increased structural vulnerability, and earlier reproductive allocation, negatively affecting long‐term survival (Körner, 2017, 2021). By contrast, slower growth in colder climates enhances structural reinforcement and carbon storage, promoting longevity and resistance to environmental stressors (Rosbakh & Poschlod, 2018). As warming accelerates growth rates, it may favor short‐lived, fast‐growing species over long‐lived ones (Chondol *et al*., 2025), potentially altering community composition and ecosystem stability (Büntgen *et al*., 2019). Understanding this grow fast–die young trade‐off and climate warming‐induced shifts is essential for predicting plant responses to climate change in extreme environments (Silvertown, 2013).

Global warming is also linked to significant changes in precipitation patterns, especially in arid mountain regions (Seneviratne, 2021). While warming drives vegetation changes in cold, humid mountains (Steinbauer *et al*., 2018), altered precipitation and its effects on soil water balance are likely more critical in dry areas (Frank *et al*., 2015). The atmosphere can hold more moisture as temperatures rise, resulting in intense and sporadic rainfall events. In arid mountains, this increases the frequency of extreme weather events, often manifesting as heavy downpours or snowfalls rather than steady precipitation (Fischer *et al*., 2021). Extreme precipitation can trigger landslides on poorly vegetated slopes, causing severe ecological disruptions and species range shifts (Lawlor *et al*., 2024). The resulting surge in soil moisture, combined with processes like frost heave, can uproot plants, adversely affecting their growth and survival (Körner, 2021). This issue is compounded by a lack of research on high‐elevation vascular plants in dry continental Asia, creating a significant knowledge gap regarding their responses to climate change.

Ladakh, an arid mountainous region in the NW Himalayas, provides a unique opportunity to study vegetation dynamics and plant growth responses to climate change (Dolezal *et al*., 2016). Its dry continental climate results in unglaciated mountains reaching 6200–6400‐m elevations, allowing plant species to thrive above 6000 m (Dvorský *et al*., 2016). Over the last century, mean temperatures in Ladakh have risen by 1.7°C (Pant *et al*., 2018), with accelerated warming noted since the 1990s, resulting in rapid glacier retreat (Schmidt & Nüsser, 2017, 2023). In addition to warming, Ladakh has also seen more frequent extreme snowfall and storm events that lead to severe flooding at lower elevation semideserts and steppes and landslides at high elevation alpine and subnival zones (Thayyen *et al*., 2013; Dolezal *et al*., 2016). Given these changing conditions, it is essential to study plant responses to contrasting drivers, such as warming, which can enhance growth and increased extreme precipitation events, which can disrupt these growth patterns.

The mechanisms driving growth and recruitment in alpine plants intrigue ecologists (Milla *et al*., 2009; Dee & Stambaugh, 2019), yet our understanding of climate drivers, especially in the Himalayas, is limited (Thakur *et al*., 2024). Research faces challenges like remote locations, short monitoring periods, and low sample replication (McCarthy, 1992). A promising approach is retrospective growth analysis, which examines annual radial increments from a plant's oldest parts (Gärtner & Schweingruber, 2013; Von Arx *et al*., 2016). This technique requires skill in identifying growth rings, particularly in wet alpine perennials with diffuse‐porous xylem (Rai *et al*., 2024), unlike semiarid subtropical species that display clear ring boundaries within ring‐porous xylem (Doležal *et al*., 2018). These records offer valuable data on past growth and biomass production, helping researchers track historical climate impacts on plant performance over decades (Francon *et al*., 2020).

This study investigates the annual recruitment and growth dynamics of *Ladakiella klimesii*, an endemic alpine forb found in the Northwest Himalayas at elevations between 5350 and 6150 m (German & Al‐Shehbaz, 2010). Conducted at 5900 m in eastern Ladakh, our research analyzes variations in plant age and growth ring width across 205 individuals to evaluate how climate change has impacted recruitment success, population structure, and growth patterns over the past 30 yr. In light of Ladakh's rapid warming, glacier retreat, and increased occurrences of extreme weather events (Schmidt & Nüsser, 2017, 2023), we explore *Ladakiella*'s sensitivity to these environmental changes using several approaches.

First, we compare ontogenetic growth patterns between plants established during colder (1998–2005) and warmer (2005–2013) periods, utilizing basal area increments derived from growth rings and the Richards growth model (Richards, 1959). This allows us to assess the effects of climate change on peak growth rates, the timing of growth deceleration (inflection point age), and maximum potential size and lifespan. Second, we employ age‐detrended and standardized individual growth and recruitment chronologies to examine how annual fluctuations in temperature and precipitation have influenced *Ladakiella*'s secondary stem growth and establishment success over the past three decades. Third, analyzing 10 yr of *in situ* microclimatic data recorded at 5900 m enabled us to assess in detail how extreme weather events, such as those in 2010 and 2015, impact plant growth and recruitment success, shedding light on the resilience of *Ladakiella* in this harsh environment. By integrating these methodologies, this study provides valuable insights into the impacts of climate warming and extreme weather events on the growth and recruitment of one of the world's highest alpine plants. While we anticipated climate warming to enhance subnival plant growth and recruitment, primarily limited by low temperatures and a short growing season, we hypothesized that extreme precipitation events would negatively affect growth and recruitment success.

## Materials and Methods

### Study species and research area

**Fig. 1:**
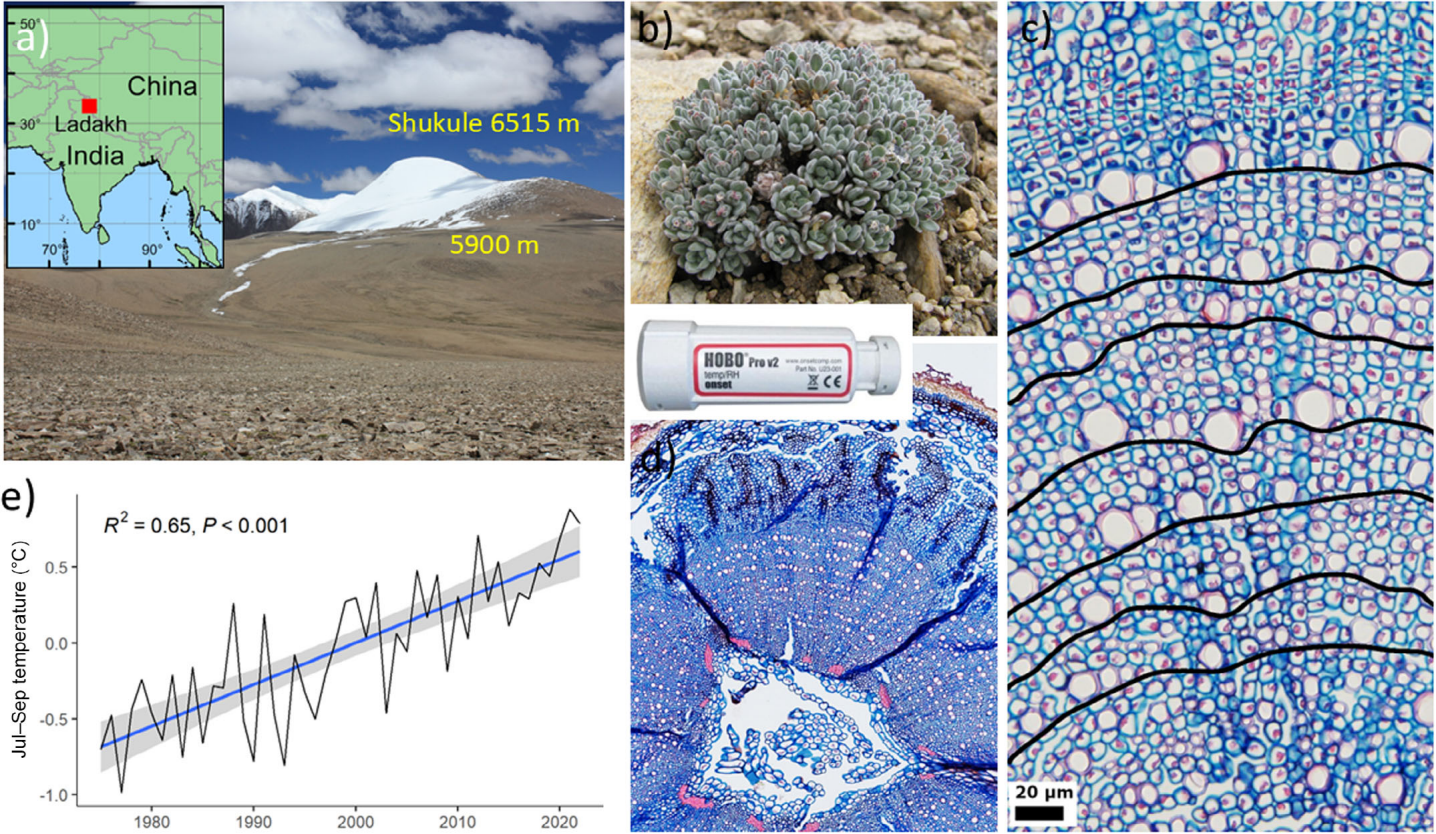
*Ladakiella klimesii*, an endemic species of Ladakh, NW Himalaya, India, was studied on the high‐elevation plateau of Shukule Peak at 5900 m (a). This perennial non‐clonal forb, with a deep tap root and compact rosette leaves (b), spans 1–30 yr with 0.03–0.1 mm radial annual growth increments (c, d), as determined from growth rings on the oldest plant tissue between the stem and root. (e) July–September temperature changes from 1975 to 2022, showing a statistically significant warming trend, with a fitted regression line and 95% confidence interval (gray band), suggesting a steady increase in temperature over the decades.

Our study examined a large population of *Ladakiella klimesii* (Al‐Shehbaz) D.A. German & Al‐Shehbaz (Brassicaceae) located at an elevation of 5900 m (32°59′N, 78°30′E; Fig. 1) on the gentle west‐facing slopes of Mount Shukule (6450 m) in Ladakh, India. This region is part of the Changthang plateau in the Northwestern Himalayas, *c*. 15 km east of Tso Moriri Lake. The area experiences arid conditions, receiving 50–250 mm of precipitation annually, mainly due to the barrier effect of the main Himalayan range, which limits summer monsoon rainfall (Pant *et al*., 2018). *Ladakiella klimesii*, also known as *Alyssum klimesii*, is a winter‐green perennial herb belonging to the Brassicaceae family (German & Al‐Shehbaz, 2010). It forms dense cushions 1–3 cm high and 2–10 cm in diameter, with a single unbranched taproot and a few lateral fine roots. The population at 5900 m receives *c*. 200 mm of annual precipitation, with a mean annual temperature of −9.84°C recorded by an *in situ* HOBO datalogger from 2008 to 2021 (Suppporting Information Figs S1–S4). Precipitation above 5000 m during the summer primarily falls as snow (Dolezal *et al*., 2016), while winter precipitation is erratic, resulting in typically thin snow layers. The local soils, derived from gneiss rock, are coarse‐grained, contain a high proportion of large gravel, and exhibit low water and organic matter content and a high pH (7–8) (Řeháková *et al*., 2017). The permanent snow line is found at altitudes of 6000–6100 m, which coincides with the highest recorded elevation of vascular plants in the area, *c*. 6150 m (Dolezal *et al*., 2016).

### Microclimatic measurements

**Fig. 2:**
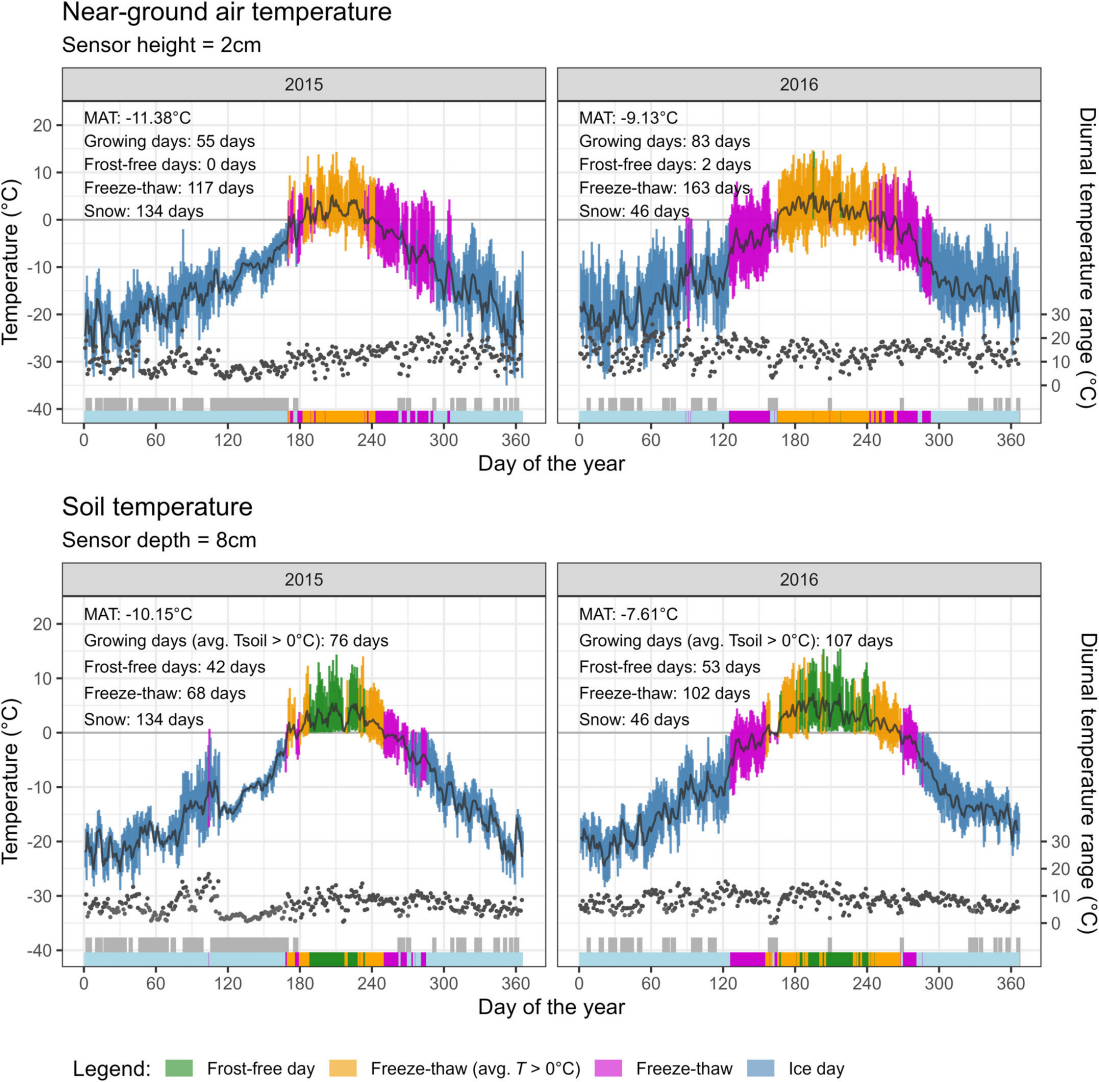
Near‐ground air and soil temperature comparisons for 2015 and 2016 show distinct thermal conditions for above‐ and belowground plant organs at 5900 m. *Ladakiella* roots, and buried stems experience 1–2 K higher mean annual temperatures (MAT) and a growing season (daily average temperature > 0°C) *c*. one‐third longer, with at least 40 frost‐free days. By contrast, aboveground organs face freezing nighttime air temperatures throughout the growing season. Reduced temperature fluctuations in late winter 2015 (April–May) suggest a deep snowpack, which shortened the growing season and limited *Ladakiella*'s growth and establishment. By contrast, 2016 provided more favorable conditions for plant growth and recruitment. A bottom shade strip highlighting snow cover is based on a range < 12°C and *T*_max_ < 1.5°C. Black dots indicate the daily temperature range.

Near‐ground air temperature was measured *in situ* using an Onset HOBO U23 Pro v.2 climate data logger (Figs 2, S5), positioned 2 cm above the ground (where the cushiony leaves of *Ladakiella* plants are primarily located) and shielded from direct sunlight. It recorded temperature and relative humidity every 2 h from 2008 until 2021 (Fig. S6). The *in situ* daily mean air temperatures showed a strong correlation (*r* = 0.96) with the daily mean temperatures from the Cru Jra v.2.1 gridded database (Harris, 2020) over the 2008–2021 period (Fig. S5). Hence, representing a reliable source of information, the gridded climate data were used to test the correspondence between climate variation and pulses of plant recruitment and radial growth changes in the sampled *Ladakiella klimesii* plants over the past 30 yr.

Since soil temperature is partly decoupled from air temperature, and belowground organs are better protected from freezing compared to aboveground stems, leaves, and inflorescences (Dolezal *et al*., 2016), we also measured soil temperature 8 cm belowground using the external temperature sensor of the HOBO U23 data logger from 2014 to 2021 (Figs S6, S7). Using on‐site near‐ground air and soil temperature data, we calculated the number of frost‐free days, representing the approximate length of the growing season, along with ice days, when temperatures remain below freezing all day, and freeze–thaw days, characterized by above‐freezing temperatures during the day and subfreezing temperatures at night.

### Plant sampling, growth, and age determination

A total of 205 individual plants were randomly selected from a 100 × 100 m area around the HOBO data logger in August 2017. Using small hand tools, the plants were excavated to preserve their root systems. After excavation, they were separated from the soil by gently shaking and rinsing in glacial water. A 1‐cm segment of the oldest part at the root collar was cut from each plant and preserved in a 50% ethanol solution to maintain tissue softness and prevent mold until lab analyses (Doležal *et al*., 2018). The remaining plant parts were divided into belowground roots and aboveground stems and leaves, placed in paper bags, and dried in an oven at 65°C until they reached a constant weight. To obtain plant age and radial growth data in the lab, several cross‐sections were cut from the root collar using a sledge microtome, stained with Astra Blue and Safranin, and permanently mounted on microscope slides with Canada Balsam (Doležal *et al*., 2018). All annual growth rings of perennial dicot plants are typically located in the root collar (Schweingruber *et al*., 2020). Microscopic images of these sections were captured using an Olympus BX53 microscope, Olympus DP73 camera, and cellSense Entry 1.9 software. The simple xylem structure of *Ladakiella klimesii* allows for easy counting of consecutive rings and measurement of annual radial growth increments, consisting of a single row of lignified earlywood vessels and four to eight rows of living parenchyma cells (Doležal *et al*., 2018). The age of each plant was estimated by counting the maximum number of rings measured along four radii from each sample.

### Ontogenetic variation in radial growth

To analyze plant ontogenetic growth trajectories and determine whether the target plants continue to grow intensively without a clear asymptote or exhibit a decline in radial growth, we applied the nonlinear Richards model (Richards, 1959) to annual basal area growth increments measured on root collar cross‐sections of each individual:

$$D_{t}=A*{\left(1+1/\delta *e^{-r*\left(t-t^{*}\right)}\right)}^{-\delta }$$

where *D*_*t*_ is the size (basal area) at time *t*, *A* is the asymptotic or maximum size, *δ* determines the more or less sigmoid shape of the curve (the shape parameter is the rate at which diameter change approaches the asymptote at inflection point), *r* defines the growth rate, *t* is the age along which the growth is studied, and t* is the age of growth inflection. The model describes early rapid growth phases and slower asymptotic approaches to a maximum size. The growth models were fitted by the nonlinear least‐squares minimizer method (R function nlxb in the package nlsr).

We examined whether plants established during the colder period of 1989–2005 (cold plants) differed in growth parameters from those established in the warmer period of 2006–2017 (warm plants) to evaluate the impact of climate change on plant development and lifespan (Fig. S8). The warm period was consistently 0.3–1.2°C warmer across all months compared to the cold period, with an average annual increase of *c*. 0.66°C. The warming is most pronounced during the transitional seasons, but is also clearly evident in summer. In July, the temperature rises by 0.42°C, and in August by 0.57°C. These increases occurred during the only months with above‐freezing temperatures, making them particularly significant for plant growth and ecological activity. Spring and autumn also show notable changes – for example, April warms by 0.92°C and October by 1.10°C—potentially extending the growing season. Since cold plants are older than warm plants, this age difference could influence the growth parameters and the observed trade‐offs between growth rate, age of growth inflection, and maximum potential size and lifespan. To address this, we conducted an additional analysis using truncated data for the older cold plants with a maximum age of 11 yr, aligning their age range with that of the younger warm plants.

### Cross‐dating and chronology development

To explore climate influences on interannual growth and recruitment variation, we first cross‐dated (i.e. attributed individual growth rings to years of their formation) ring width series visually and statistically using the Past5 software (SCIEM, Brunn/Geb., Austria; www.sciem.com). The best cross‐dated samples from 171 individuals were included in a standard chronology that retains autocorrelation both at the sample and chronology level (Fig. 4). We removed the age trend from the cross‐dated series by applying a negative exponential curve, allowing for the positive slopes using the dplr package (Bunn, 2008) in R (R Core Team, 2023). A bi‐weight robust mean of the detrended series was calculated to construct the standard chronology. The reliability of the chronology was tested by statistical assessments based on the percentage of parallel variation (GLK—Gleichläufigkeit). GLK is a standard test used to estimate the signal strength of individual series and the suitability of a chronology for past climate reconstruction; GLK values > 60% are considered sufficient for reliable reconstruction of past climate (Esper & Gärtner, 2001; Buras & Wilmking, 2015). The mean GLK of the series used for chronology development reached 65.43%. In addition to GLK, the measured series' mean correlation with standard chronology was 0.42. Hence, mean series correlation with chronology alongside GLK indicates a robust standard signal among the used series and, thus, the developed chronology.

### Correlation analysis with daily climatic data

We analyzed correlations between the standard chronology and daily climatic data to identify the key periods and climatic factors influencing the growth of *Ladakiella klimesii*. For this analysis, we utilized temperature and precipitation data from the gridded Cru Jra v.2.1 database (Harris, 2020). Unlike traditional monthly correlations, this method is not constrained by calendar months, allowing us to gain insights into seasonal growth activity peaks and their limiting climatic factors (Jevšenak, 2020). We employed the R package dendrotools (Jevšenak & Levanič, 2018), specifically utilizing the function daily_response(). This function employs a moving window of varying widths to calculate correlation coefficients between an aggregated climate variable and the standard chronology. We computed daily correlations for window widths ranging from 40 to 270 consecutive days, using a sliding window that begins on January 1 before growth ring formation and ends on December 31 of the year the ring was formed. The selected range of consecutive days was intended to avoid random correlations due to overly short windows while capturing the potential impacts of seasonal climate, with an upper limit set at 270 d.

## Results

### Climate variation

Long‐term meteorological records from Ladakh indicate a significant rise in temperature and fluctuations in precipitation since the 1990s (Figs 1, S1–S4). *In situ* near‐ground air temperature data from our study area at 5900 m elevation, covering 2008–2021, confirm this trend (Fig. S6). There is considerable variation in growing season length across the years, with the shortest growing seasons occurring in colder years. Overall, warmer years exhibit extended growing seasons, fewer snow cover days, and increased freeze–thaw variability (Figs 2, S6). Between 2008 and 2021, the growing season ranged from 55 to 89 frost‐free days based on near‐ground air temperatures (Fig. S6). A comparable analysis utilizing soil temperature data from 2014 to 2021 revealed an extended frost‐free period lasting between 76 and 106 d (Fig. S7). Additionally, the mean growing season temperature based on these soil measurements increased from 2.42°C in 2009 to 3.45°C in 2021. The growing season in 2009, 2010, and 2015 lasted only 55–56 d. These years were characterized by long snow cover durations, such as 221 and 204 d in 2009 and 2010, and minimal frost‐free days. By contrast, the longest growing seasons occurred in 2016, 2018, and 2021, with growing periods extending from 83 to 89 d (Fig. 2). These warmer years point toward climate shifts that result in earlier snowmelt, increased temperature fluctuations, and prolonged periods of above‐freezing temperatures. For example, 2016 had only 46 d of snow cover compared to 221 in 2009, further demonstrating the impact of warmer conditions on seasonal transitions.

A comparison of soil and near‐ground air temperatures revealed large differences in the thermal conditions experienced by aboveground and belowground plant organs (Fig. 2). In the soil, *Ladakiella* roots and buried stem bases benefit from warmer conditions compared to stems, leaves, and flowers, with mean annual temperatures being 1–2 K higher and the growing season extended by *c*. one‐third, providing at least 40 frost‐free days. Freezing near‐ground air temperatures occurred on most nights during the growing season (97%), with mean daily minimum temperatures ranging from −1.96°C to −5.29°C and absolute minimum temperatures between −5.41°C and −11.29°C. During the growing season, maximum daily air temperatures ranged from 8.31°C to 14.79°C, with absolute highs reaching between 13.32°C and 25.04°C. In winter, mean air temperatures during the coldest months of December and January drop to −15.44°C, with minimums reaching as low as −36.1°C. *Ladakiella* also regularly experiences snowfall during the growing season, with an average of 17.83 d of nighttime snowfall, which typically melt by the following day.

### Population age and size structure

**Fig. 3:**
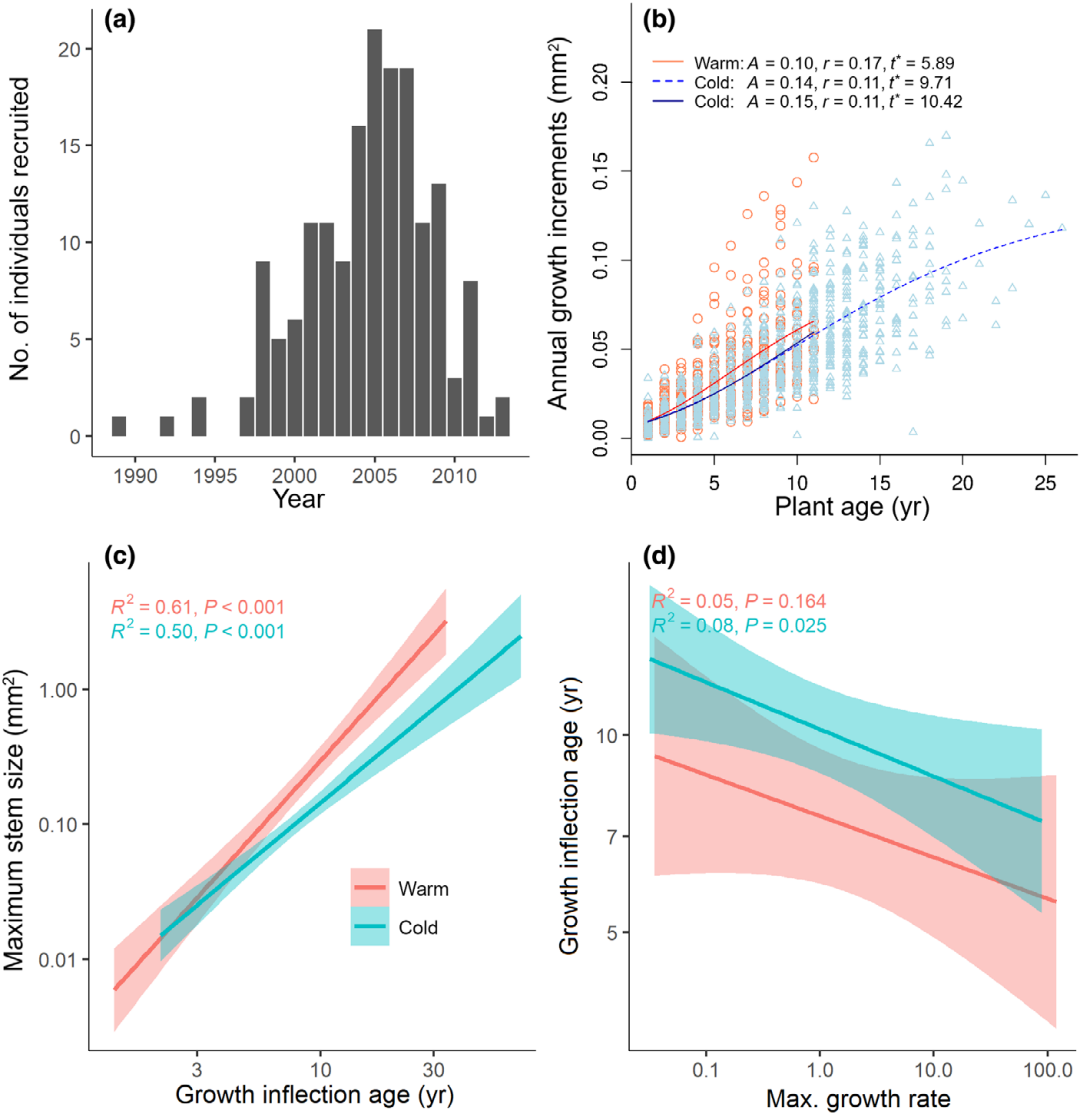
The growth characteristics and age distributions of *Ladakiella klimesii*. (a) Age distributions in the subnival zone at 5900 m. (b) Ontogenetic stem basal area increments measured on a root collar (mm^2^), fitted by the Richards growth model, for plants established in colder and warmer periods during the past 30 yr. For cold plants (blue lines), growth parameters are shown for both non‐truncated (dashed line) and truncated datasets (solid line) to match the age range of warm plants. (c) Relationships between growth parameter estimates from the Richards growth model for cold and warm plants. (d) Relationships between the asymptotic stem size (*A*), the maximum growth rate (*r*), and the timing of growth deceleration (t*, growth inflection age).

The ages of the plants varied from 5 to 29 yr (Fig. 3a), with an average age of 13.6 ± 4.05 yr (mean ± SD). The root collars ranged from 0.42 to 3.28 mm, with a mean diameter of 1.67 mm ± 0.54. Their total dry biomass varied between 0.109 and 5.425 g, with an average weight of 1.035 ± 0.96 g. The plants allocated the majority of their biomass to leaves (67.9 ± 13.3%), followed by stems (19.7 ± 12.5%) and roots (12.2 ± 6.6%). Annual radial growth increments varied from 5.73 to 116.1 μm, with a mean of 26.63 ± 5.52 μm. The age distribution of the plants followed a symmetric pattern, with most individuals 10–15 yr old.

### Ontogenetic growth variations

Of the 171 plants examined, 95.3% of individuals displayed basal area growth at the root collar that was well‐fitted by the sigmoidal Richards model (Fig. S8). A comparison of the model parameters between plants established during colder periods (1989–2005, cold plants) and those established in warmer periods (2006–2013, warm plants) showed significant differences in the asymptotic size (*A*, *P* = 0.012; Fig. 3b), the maximum growth rate (*r*, *P* = 0.041), the age of maximum growth rate (*P* < 0.001), and the age of growth inflection (t*, *P* = 0.001). Warm plants had 35.3% faster growth and reached their maximum growth rates 5.61 yr earlier than cold plants. However, cold plants maintained exponential growth for longer, reaching the growth deceleration (inflection age) on average 4.53 yr later (10.39 yr vs 5.89 yr). Cold plants with slower growth and lower maximum growth rates achieved larger stem diameters, as indicated by positive correlations between the maximum (asymptotic) size and age of growth inflection (Fig. 3c) and negative correlations between maximum growth rate and growth inflection age (Fig. 3d). An additional analysis using truncated data for cold plants, aligning their age range with that of warm plants, showed no significant differences in growth parameters between truncated and non‐truncated cold plants (*P* > 0.05; Fig. 3b). This indicates that the observed differences in ontogenetic growth and lifespan between plants originating in contrasting climatic conditions are not biased by the length of growth intervals.

### Climate influence on growth and recruitment

**Fig. 4:**
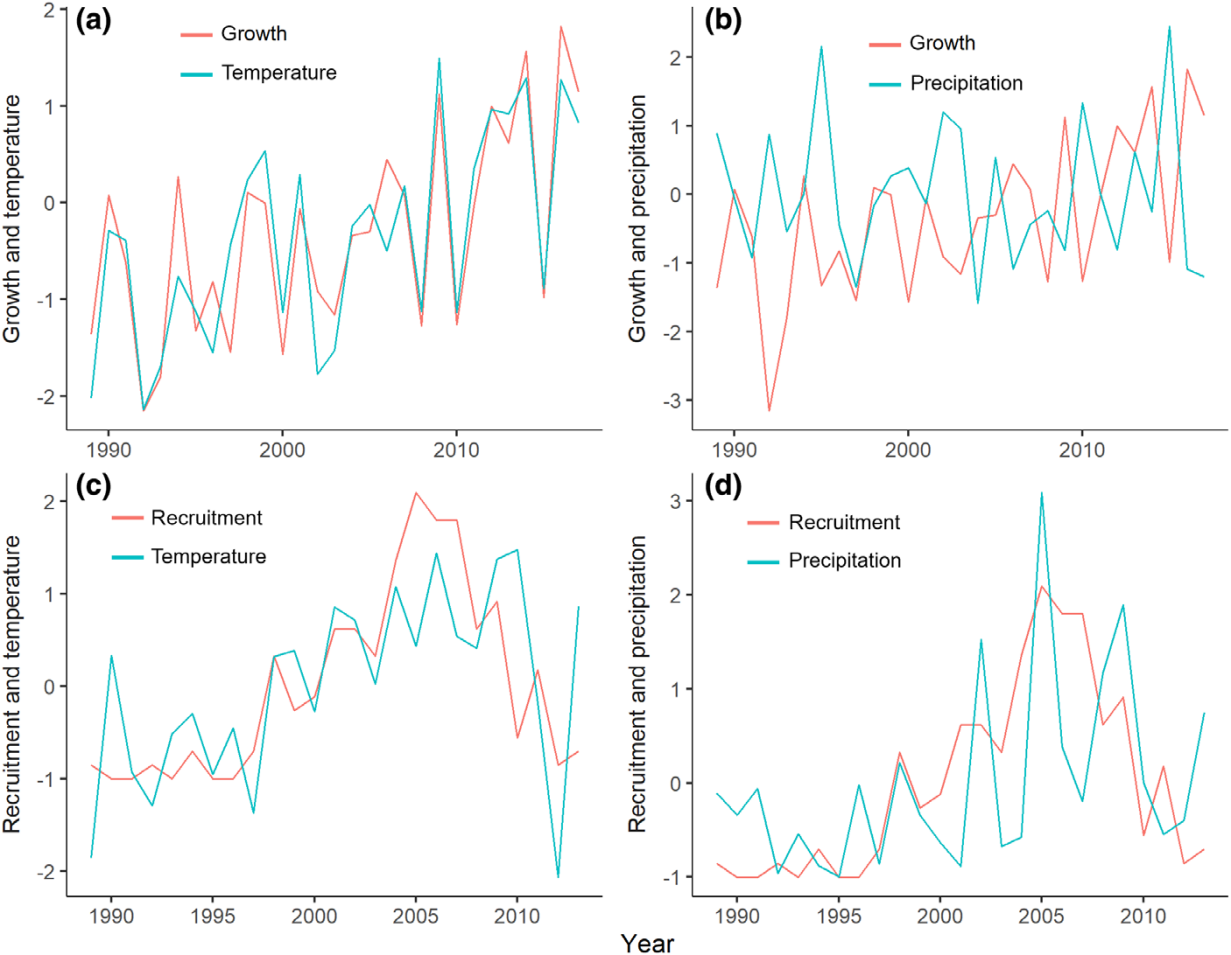
Correlations between biological metrics of *Ladakiella klimesii* (growth and recruitment) and climatic variables (temperature and precipitation) were analyzed using Z‐scores of standard chronologies (green lines). (a) Growth was most strongly influenced by temperatures between June 29 and October 4 (98 d, *r* = 0.911, *P* < 0.01). (b) By contrast, winter precipitation had a negative effect on growth (December 30–March 11, *r* = −0.623, *P* < 0.01). (c) Recruitment showed the highest positive correlation with winter temperatures (the previous year, October 17–July 12, *r* = 0.63, *P* < 0.01) and (d) with the current year precipitation from September 9 to October 27 (*r* = 0.529, *P* < 0.01).

Over the past 30 yr, the radial growth of *Ladakiella* exhibited a marked increase with notable fluctuations that align closely with rising summer temperatures (Fig. 4a). A strong positive correlation exists between the two variables (*r* = 0.911, *P* < 0.01), with their trends closely mirroring each other, especially after 2000. This suggests that higher summer temperatures are closely linked to enhanced growth. The period between June 29 and October 4 (98 d) was identified as the most effective time frame for explaining interannual variations in radial growth (Fig. S9). By contrast, precipitation shows an inverse relationship with growth (*r* = −0.623, *P* < 0.01) (Fig. 4b). Specifically, increased winter precipitation is associated with lower growth values, indicating that excessive snowfall during the winter may negatively affect subsequent growth. The winter precipitation recorded from December 30 of the previous year to March 11 (73 d) was found to be most closely related to interannual variations in *Ladakiella* growth (Fig. S10).

The recruitment showed the strongest positive correlation with temperatures from October 17 of the previous year to July 12 of the current year (*r* = 0.63, *P* < 0.01; Fig. 4c). Both variables increased notably after the mid‐1990s, with a peak in recruitment around the early 2000s, supporting the prominent role of winter temperature in influencing establishment success. Recruitment also positively correlated with autumn precipitation from September 9 to October 27 (*r* = 0.529, *P* < 0.01; Fig. 4d). While recruitment and precipitation exhibit some synchronous peaks, particularly in the early 2000s, there are periods where recruitment increases while precipitation remains stable or declines, suggesting a more complex interaction between these variables.

## Discussion

Using herbchronology, we assessed the long‐term growth and recruitment dynamics of *Ladakiella klimesii*, an endemic alpine forb of the West Himalayas, over the past 30 yr. Several key findings emerged from studying the world's highest‐occurring plants at 5900 m. Radial growth closely correlates with summer temperature (*r* = 0.911), with higher temperatures enhancing growth, while winter precipitation negatively impacts it. By contrast, recruitment benefits from warmer winters and springs and increased autumn precipitation. These results align with broader research on alpine and arctic plant responses to climate change (Büntgen *et al*., 2015; Myers‐Smith & Hik, 2018; Dee & Stambaugh, 2019), which highlights the rapid ecological shifts in warming mountain ecosystems (Anderson *et al*., 2020; Francon *et al*., 2020; Rai *et al*., 2024). Climate warming also alters *Ladakiella* growth trajectories by influencing the balance between growth rate, size, and lifespan. Plants established in colder periods grow more slowly and live longer, whereas those recruited in warmer conditions grow faster but have shorter lifespans, suggesting a trade‐off between rapid growth and longevity that is susceptible to affecting long‐term population stability (Rosbakh & Poschlod, 2018; Büntgen *et al*., 2019; Chondol *et al*., 2024). Increased precipitation fluctuations and heavy snowfalls in late winter and summer further disrupt plant growth and recruitment due to intensified soil disturbances, such as needle ice formation and frost upheaval (Dolezal *et al*., 2016). These findings underscore the sensitivity of Himalayan alpine plants to climate warming and shifting precipitation patterns, demonstrating the utility of herbchronology in monitoring ecological and climatic changes in these fragile mountain ecosystems.

### Climate‐driven growth dynamics

An analysis of growth and recruitment drivers in the alpine cushion plant *Ladakiella klimesii* in the cold‐arid Ladakh region highlights significant climate influences. By cross‐dating growth ring widths and analyzing them with daily climate data, we could accurately assess the impact of climate change on radial stem growth, an approach rarely applied to alpine cushion plants (Rai *et al*., 2024). Unlike alpine plants in wetter mountain environments, where prolonged snow cover often results in missing growth rings (Schweingruber *et al*., 2020), *Ladakiella* demonstrated continuous growth ring formation. This consistency allows for precise alignment with calendar years to reveal robust climatic signals within its xylem chronology. The xylem structure of *Ladakiella* is relatively simple, consisting of a single row of earlywood vessels with lignified walls and four to eight rows of living parenchymatic cells (Fig. 1). These cells correspond to 55–90 growing season days (July–September) with temperatures above zero, requiring 7–10 d for new cell formation at 2–3°C, compared to just 24 h at 20°C (Körner, 2021). These constraints make *Ladakiella* highly sensitive to annual summer temperature variability (correlation *r* = 0.911 for 1989–2017), with recent warming reducing low‐temperature stress, enhancing growth, and promoting seedling regeneration. Over the past 30 yr, *Ladakiella* has maintained consistent growth at mean growing season temperatures of 2–3°C, averaging an annual radial growth of 0.026 mm, demonstrating its resilience to climate variability.

Like trees adapting to climate change (Büntgen *et al*., 2019), *Ladakiella* plants exhibited rapid early growth in warmer climates, with growth increments increasing steeply and reaching a maximum age of *c*. 10 yr. Individual growth trajectories vary, but the overall trend suggests accelerated development in warmer climates. By contrast, plants in colder climates grow more gradually over a longer lifespan, with growth increments rising steadily beyond 20 yr. The greater variation in individual growth patterns indicates a more prolonged growth phase. These results highlight a growth–longevity trade‐off, where faster early growth in warmer conditions may come at the cost of reduced lifespan (Büntgen *et al*., 2019), while slower growth in colder environments enhances long‐term persistence (Bigler & Veblen, 2009; Chondol *et al*., 2023). The findings align with climate‐driven shifts in plant growth rate, suggesting that warming accelerates early development but may ultimately limit plant longevity and maximum size (Silvertown, 2013; Rosbakh & Poschlod, 2018).

### Impact of precipitation on radial growth of *Ladakiella* plants

We found that higher daily temperatures from early July to late September boosted radial growth increments, while increased snow precipitation from early January to mid‐March hindered them. The effects of precipitation in higher elevation, colder habitats are complex, largely due to the contrasting impacts of snow (Campbell, 2019; Körner, 2021). Increased winter precipitation typically leads to thicker snow cover, which protects soil and plants from freezing during winter (Körner *et al*., 2019) and promotes microbial activity, resulting in more plant‐available nutrients (Schmidt & Lipson, 2004). While increased snow cover can buffer plants from frost damage during the spring (Rixen *et al*., 2010; Choler, 2018; Krab *et al*., 2018), it may also delay snowmelt and shorten the limited growing season (Dolezal *et al*., 2020), increase the size of permanent snowfields, and obscure habitable areas (Stöcklin & Bäumler, 1996). Furthermore, late snowmelt caused by a thick snowpack may lead to soil disturbances due to solifluction, presenting challenges for vascular plants adapted to the arid and largely unvegetated soils of cold Himalayan deserts, such as *Ladakiella klimesii* (Dvorský *et al*., 2016).

The *Ladakiella* plants studied on the Changtang plateau of eastern Ladakh are largely cut off from major precipitation sources due to geographic barriers. The Karakoram range to the northwest blocks winter westerlies, while the Great Himalayas to the south obstruct the summer monsoon. The winter Western Disturbance, bringing moisture from westerly winds across regions like the Mediterranean and Caspian Seas, contributes *c*. 65% of annual precipitation, especially in the Karakoram Mountains (Pant *et al*., 2018), but this system was not stable in the past decades (Dar, 2023). From July to September, the summer monsoon delivers moisture via easterly winds from the Bay of Bengal but rarely crosses the Main Himalayan Range. This isolation and limited glacier extent in the rainshadow of the central mountain ranges have pushed *Ladakiella* and *about* six other vascular plant species to some of the highest elevations on Earth, reaching up to 6200 m (Dvorský *et al*., 2011; Angel *et al*., 2016). Some alpine plants are thought to grow even higher on Mount Everest (6400 m), based on historical records from the 1930s and 1950s (Dentant, 2018), but these claims remain unverified in recent studies. The poor glaciation and presence of plants at such high elevations in Ladakh stem from the minimal influence of winter westerlies, which do not reach the Changtang plateau, necessitating adaptations for winter survival without snow cover. Consequently, years of heavy snowfall in spring and summer can negatively impact plant recruitment and growth, such as in the spring of 2015. Likewise, extreme summer snowfall from the monsoon, as seen in the summer of 2010 (Thayyen *et al*., 2013), caused subnival plants to suffer increased mortality (Dolezal *et al*., 2016). We observed this phenomenon caused by intensified soil disturbances linked to needle ice formation, frost upheaval, meltwater, and solifluction.

### Impact of temperature and precipitation on the recruitment of *Ladakiella* plants

With minimal winter snow cover for insulation, sudden spring and summer snow accumulation is detrimental to *Ladakiella* at 5900 m. A significant aspect of climate change in this region is the increasing frequency of extreme precipitation events, such as those in the spring of 2015 and summer of 2010, which negatively impacted vascular plants in the cold‐arid Himalayas and caused casualties in Ladakh (Thayyen *et al*., 2013; Dvorský *et al*., 2016). Dvorský *et al*. (2016) found that while many transplanted species survived at 6000 m for several years, none persisted after extreme snowfalls, highlighting the influence of episodic climatic events on their upper elevational limits. Species better adapted to repeated soil freezing and thawing conditions showed significantly higher survival rates, although wet soils combined with freezing temperatures hindered seedling establishment and mature plant survival (Dolezal *et al*., 2016). Hence, increased water input may negate the benefits of warming and longer growing seasons, potentially leading to population declines (Dolezal *et al*., 2021). Likewise, *Ladakiella* recruitment in the subnival zone did not peak during the warmest decade, likely due to extreme snowfall events, as evidenced by aging populations and fewer younger individuals since 2010, similar to *Potentilla pamirica* (Dolezal *et al*., 2021). However, *Ladakiella* populations are unlikely to disappear due to recruitment pulses following warm winters and springs, with autumn snow cover providing protection for seedlings. This suggests a reliance on fresh seeds and a soil seed bank, with non‐clonal species like *Ladakiella* responding rapidly to favorable conditions, unlike clonal shrubs that may exhibit delayed recruitment (Büntgen *et al*., 2015).

### Developmental controls vs climatic variation

*Ladakiella* in the Eastern Ladakh mountains (Changtang, Tibetan Plateau) endures extreme climatic variability, with minimal winter snow cover and frequent growing‐season snowfall creating freeze–thaw cycles (Dvorský *et al*., 2016; Miehe *et al*., 2019). While alpine plants often adopt conservative developmental controls to buffer against annual fluctuations (Körner, 2023), *Ladakiella*'s stem radial growth closely tracks summer temperature variation. Typically, long‐lived species in stable environments rely more on genetic control (internal clock) over growth and phenology, whereas short‐lived pioneers in variable habitats adopt opportunistic strategies (Chuine, 2010). In our study, wind‐exposed plateaus (5700–6150 m) with minimal snow protection should favor conservative phenology, yet *Ladakiella* shows high responsiveness to annual temperature shifts. This suggests that its root collars, where growth rings were measured, are insulated by soil, allowing belowground structures to be more climate‐responsive than exposed leaves, buds, and flowers. Temperature records from 5900 m indicate near‐ground air temperatures drop below freezing (−5°C) for 5–10 h nightly, while soil temperatures remain above zero. This highlights the role of microclimatic buffering in modulating plant organ responses to environmental stress.

*Ladakiella*'s ability to persist for decades and buffer environmental stress is likely supported by its high storage capacity. Its stems and roots contain 3–4% nitrogen (Dolezal *et al*., 2016) and 20–30% dry weight in nonstructural carbohydrates, primarily fructans and soluble sugars – the highest among 11 perennial species studied across 4500–6000 m in the Western Himalayas (Chlumská *et al*., 2022). This is linked to its low structural dry matter density, with over 80% of stem tissue composed of living parenchyma cells optimized for carbohydrate storage (Doležal *et al*., 2018). These reserves likely play a key role in mitigating environmental stress. Moreover, despite its sensitivity to climate variability, *Ladakiella*'s growth is likely moderated by genetic controls (internal clock) over seasonal activity. Its strongest growth correlation with summer temperatures (June 29–October 4; 98 d) suggests avoiding early summer growth, even when maximum daytime temperatures are possibly conducive to growth. This may be due to much lower minimum temperatures in June (Fig. 2), influencing phenological transitions such as dormancy, bud break, flowering, and senescence (Körner *et al*., 2019). These genetically regulated cues ensure reproduction in favorable years while supporting long‐term survival through sustained stem growth (Forrest & Miller‐Rushing, 2010).

*Ladakiella* can thus be regarded as a long‐lived pioneer species, characterized by its lifespan of over 30 yr (Schweingruber *et al*., 2020), colonization potential (Ruka *et al*., 2023), and yr‐to‐yr growth and recruitment shaped by climatic fluctuations (Fig. 4). It occupies an intermediate ecological position, displaying pioneer traits by rapidly colonizing newly exposed subnival zones, particularly areas uncovered by glacier retreat (Angel *et al*., 2016; Dolezal *et al*., 2016; Ruka *et al*., 2023). In contrast to short‐lived pioneer species that typically establish rapidly but shed their aboveground tissues each year (Dolezal *et al*., 2024), *Ladakiella* persists for over three decades, forming compact cushion canopies with durable aboveground stems (Klimešová *et al*., 2011).

### Conclusions

The analysis of *Ladakiella klimesii* reveals that its radial growth is highly sensitive to summer temperatures, with warmer conditions significantly enhancing growth, while increased winter precipitation inhibits growth, presumably through delays in snowmelt and a shortened growing season. Recruitment is favored by warmer winters and springs in combination with autumn snow cover, underscoring how different climatic factors uniquely influence various life stages. Additionally, the study highlights a distinct trade‐off between growth rate and longevity: Plants established during warmer periods tend to grow rapidly but have shorter lifespans, whereas those emerging in colder conditions grow more slowly yet persist longer, a dynamic that could affect long‐term population stability. Extreme late winter and summer snowfall events further disrupt plant growth and seedling establishment through soil disturbances and frost heave, emphasizing the vulnerability of these high‐altitude species to climate variability. Finally, the ability of *Ladakiella* to modulate its responses through insulated belowground structures demonstrates the critical role of microclimatic buffering in enabling this long‐lived pioneer species to colonize newly exposed areas above 6000 m despite harsh alpine conditions.

## Competing interests

None declared.

## Author contributions

JD and VJ conceived the research. JD and MM collected the plants. VJ and HS conducted the growth and age analyses. JA and PF conducted growth–climate analyses. VJ, JD and ATR wrote the first draft of the manuscript, and all authors approved the final manuscript before submission.

## Disclaimer

The New Phytologist Foundation remains neutral with regard to jurisdictional claims in maps and in any institutional affiliations.

## Supporting information


**Fig. S1** Monthly average temperature trends from 1975 to 2022 at 5900 m elevation in the Himalayan subnival zone.
**Fig. S2** Century‐scale monthly temperature trends from 1900 to 2022.
**Fig. S3** Monthly precipitation trends from 1975 to 2022.
**Fig. S4** Long‐term trends in monthly precipitation sums from 1900 to 2022.
**Fig. S5** Correlation between *in situ* and CRU gridded daily temperatures.
**Fig. S6** Interannual near‐ground temperature variations (2009–2021) at 5900 m.
**Fig. S7** Interannual soil temperature dynamics (2014–2021) at 8 cm depth.
**Fig. S8** Ontogenetic growth changes in warmer and colder climates.
**Fig. S9** Correlations between growth and temperature and precipitation.
**Fig. S10** Correlations between recruitment and temperature and precipitation.
Please note: Wiley is not responsible for the content or functionality of any Supporting Information supplied by the authors. Any queries (other than missing material) should be directed to the *New Phytologist* Central Office.

## Data Availability

Data available from the Zenodo repository doi: 10.5281/zenodo.15330848 (Dolezal, 2025).

## References

[nph70206-bib-0001] Anderson K , Fawcett D , Cugulliere A , Benford S , Jones D , Leng R . 2020. Vegetation expansion in the subnival Hindu Kush Himalaya. Global Change Biology 26: 1608–1625. 31918454 10.1111/gcb.14919 PMC7078945

[nph70206-bib-0002] Angel R , Conrad R , Dvorsky M , Kopecky M , Kotilínek M , Hiiesalu I , Schweingruber F , Doležal J . 2016. The root‐associated microbial community of the world's highest growing vascular plants. Microbial Ecology 72: 394–406. 27245598 10.1007/s00248-016-0779-8 PMC4937074

[nph70206-bib-0003] Aubert S , Boucher F , Lavergne S , Renaud J , Choler P . 2014. 1914–2014: a revised worldwide catalogue of cushion plants 100 years after Hauri and Schröter. Alpine Botany 124: 59–70.

[nph70206-bib-0004] Bigler C , Veblen TT . 2009. Increased early growth rates decrease longevities of conifers in subalpine forests. Oikos 118: 1130–1138.

[nph70206-bib-0005] Bonanomi G , Idbella M , Allegrezza M , Tesei G . 2023. Dieback of the cushion plant Silene acaulis at its southern limit of distribution in the Apennines. Alpine Botany 133: 57–62.

[nph70206-bib-0007] Bunn AG . 2008. A dendrochronology program library in R (dplR). Dendrochronologia 26: 115–124.

[nph70206-bib-0008] Büntgen U , Hellmann L , Tegel W , Normand S , Myers‐Smith I , Kirdyanov AV , Nievergelt D , Schweingruber FH . 2015. Temperature‐induced recruitment pulses of Arctic dwarf shrub communities. Journal of Ecology 103: 489–501.

[nph70206-bib-0009] Büntgen U , Krusic PJ , Piermattei A , Coomes DA , Esper J , Myglan VS , Kirdyanov AV , Camarero JJ , Crivellaro A , Körner C . 2019. Limited capacity of tree growth to mitigate the global greenhouse effect under predicted warming. Nature Communications 10: 2171. 10.1038/s41467-019-10174-4 PMC6520339 31092831

[nph70206-bib-0010] Buras A , Wilmking M . 2015. Correcting the calculation of Gleichläufigkeit. Dendrochronologia 34: 29–30.

[nph70206-bib-0011] Callaway RM . 2007. Positive interactions and interdependence in plant communities. Dordrecht, the Netherlands: Springer.

[nph70206-bib-0012] Campbell DR . 2019. Early snowmelt projected to cause population decline in a subalpine plant. Proceedings of the National Academy of Sciences, USA 116: 12901–12906. 10.1073/pnas.1820096116 PMC6600911 31182600

[nph70206-bib-0013] Centenaro G , Petraglia A , Carbognani M , Piotti A , Hudek C , Büntgen U , Crivellaro A . 2023. The oldest known clones of Salix herbacea growing in the Northern Apennines, Italy are at least 2000 years old. American Journal of Botany 110: e16243. 37755870 10.1002/ajb2.16243

[nph70206-bib-0014] Chlumská Z , Liancourt P , Hartmann H , Bartoš M , Altman J , Dvorský M , Hubáček T , Borovec J , Čapková K , Kotilínek M *et al*. 2022. Species‐ and compound‐specific dynamics of nonstructural carbohydrates toward the world's upper distribution of vascular plants. Environmental and Experimental Botany 201: 104985.

[nph70206-bib-0015] Choler P . 2018. Winter soil temperature dependence of alpine plant distribution: implications for anticipating vegetation changes under a warming climate. Perspectives in Plant Ecology, Evolution and Systematics 30: 6–15.

[nph70206-bib-0016] Chondol T , Klimeš A , Altman J , Čapková K , Dvorský M , Hiiesalu I , Jandová V , Kopecký M , Macek M , Řeháková K *et al*. 2023. Habitat preferences and functional traits drive longevity in Himalayan high‐mountain plants. Oikos 2023: e010073.

[nph70206-bib-0017] Chondol T , Klimeš A , Hiiesalu I , Altman J , Čapková K , Jandová V , Kopecký M , Macek M , Řeháková K , Doležal J . 2025. Contrasting habitat associations and ecophysiological adaptations drive interspecific growth differences among Himalayan high‐mountain plants. Annals of Botany: mcaf014. doi: 10.1093/aob/mcaf014. 39868547 PMC12259540

[nph70206-bib-0018] Chondol T , Korznikov KA , Doležal J . 2024. Ecological significance of marcescence in Himalayan plants: why is standing dead phytomass more important in demanding, resource‐limited environments? Functional Ecology 38: 942–954.

[nph70206-bib-0019] Chuine I . 2010. Why does phenology drive species distribution? Philosophical Transactions of the Royal Society of London. Series B: Biological Sciences 365: 3149–3160. 20819809 10.1098/rstb.2010.0142 PMC2981946

[nph70206-bib-0020] Dar J . 2023. Western disturbances alter the trend of winter precipitation and its extremes over Northwest Himalayas: Kashmir Himalaya. Environmental Science and Pollution Research 30: 83439–83451. 37344715 10.1007/s11356-023-28263-9

[nph70206-bib-0021] Dee JR , Stambaugh MC . 2019. A new approach towards climate monitoring in Rocky Mountain alpine plant communities: a case study using herb‐chronology and Penstemon whippleanus. Arctic, Antarctic, and Alpine Research 51: 84–95.

[nph70206-bib-0022] Dentant C . 2018. The highest vascular plants on Earth. Alpine Botany 128: 97–106.

[nph70206-bib-0080] Dolezal J. 2025. Ladakiella klimesii growth and recruitment data over the past 30 years in western Himalaya together with local temperature and precipitation data [Data set]. Zenodo. doi: 10.5281/zenodo.15330848.

[nph70206-bib-0023] Dolezal J , Chondol T , Chlumská Z , Altman J , Čapková K , Dvorský M , Fibich P , Korznikov KA , Ruka AT , Kopecký M *et al*. 2024. Contrasting biomass allocations explain adaptations to cold and drought in the world's highest‐growing angiosperms. Annals of Botany 134: 401–414. 38407819 10.1093/aob/mcae028 PMC11341669

[nph70206-bib-0024] Doležal J , Dvorský M , Börner A , Wild J , Schweingruber FH . 2018. Anatomy, age and ecology of high mountain plants in Ladakh, the Western Himalaya. Cham, Switzerland: Springer.

[nph70206-bib-0025] Dolezal J , Dvorsky M , Kopecky M , Liancourt P , Hiiesalu I , Macek M , Altman J , Chlumska Z , Rehakova K , Capkova K *et al*. 2016. Vegetation dynamics at the upper elevational limit of vascular plants in Himalaya. Scientific Reports 6: 24881. 27143226 10.1038/srep24881 PMC4855180

[nph70206-bib-0026] Dolezal J , Jandova V , Macek M , Mudrak O , Altman J , Schweingruber FH , Liancourt P . 2021. Climate warming drives Himalayan alpine plant growth and recruitment dynamics. Journal of Ecology 109: 179–190.

[nph70206-bib-0027] Dolezal J , Kopecky M , Dvorsky M , Macek M , Rehakova K , Capkova K , Borovec J , Schweingruber F , Liancourt P , Altman J . 2019. Sink limitation of plant growth determines tree line in the arid Himalayas. Functional Ecology 33: 553–565.

[nph70206-bib-0028] Dolezal J , Kurnotova M , Stastna P , Klimesova J . 2020. Alpine plant growth and reproduction dynamics in a warmer world. New Phytologist 228: 1295–1305. 32632948 10.1111/nph.16790

[nph70206-bib-0029] Duchicela SA , Cuesta F , Tovar C , Muriel P , Jaramillo R , Salazar E , Pinto E . 2021. Microclimatic warming leads to a decrease in species and growth form diversity: insights from a tropical alpine grassland. Frontiers in Ecology and Evolution 9: 673655.

[nph70206-bib-0030] Dvorský M , Chlumská Z , Altman J , Čapková K , Řeháková K , Macek M , Kopecký M , Liancourt P , Doležal J . 2016. Gardening in the zone of death: an experimental assessment of the absolute elevation limit of vascular plants. Scientific Reports 6: 24440. 27071305 10.1038/srep24440 PMC4829891

[nph70206-bib-0031] Dvorský M , Doležal J , De Bello F , Klimešová J , Klimeš L . 2011. Vegetation types of East Ladakh: species and growth form composition along main environmental gradients: vegetation of East Ladakh. Applied Vegetation Science 14: 132–147.

[nph70206-bib-0032] Dvorský M , Doležal J , Kopecký M , Chlumská Z , Janatková K , Altman J , De Bello F , Řeháková K . 2013. Testing the stress‐gradient hypothesis at the roof of the world: effects of the cushion plant *Thylacospermum caespitosum* on species assemblages. PLoS ONE 8: e53514. 23326446 10.1371/journal.pone.0053514 PMC3542354

[nph70206-bib-0033] Esper J , Gärtner H . 2001. Interpretation of tree‐ring chronologies (Interpretation von Jahrringchronologien). 277–288.

[nph70206-bib-0034] Ferrarini A , Alsafran MHSA , Dai J , Alatalo JM . 2019. Improving niche projections of plant species under climate change: silene acaulis on the British Isles as a case study. Climate Dynamics 52: 1413–1423.

[nph70206-bib-0035] Fischer EM , Sippel S , Knutti R . 2021. Increasing probability of record‐shattering climate extremes. Nature Climate Change 11: 689–695. 10.1038/s41558-021-01092-9 PMC7617090 39650282

[nph70206-bib-0036] Forrest J , Miller‐Rushing AJ . 2010. Toward a synthetic understanding of the role of phenology in ecology and evolution. Philosophical Transactions of the Royal Society of London. Series B: Biological Sciences 365: 3101–3112. 20819806 10.1098/rstb.2010.0145 PMC2981948

[nph70206-bib-0037] Francon L , Corona C , Till‐Bottraud I , Carlson BZ , Stoffel M . 2020. Some (do not) like it hot: shrub growth is hampered by heat and drought at the alpine treeline in recent decades. American Journal of Botany 107: 607–617. 32239494 10.1002/ajb2.1459

[nph70206-bib-0038] Frank D , Reichstein M , Bahn M , Thonicke K , Frank D , Mahecha MD , Smith P , Van Der Velde M , Vicca S , Babst F *et al*. 2015. Effects of climate extremes on the terrestrial carbon cycle: concepts, processes and potential future impacts. Global Change Biology 21: 2861–2880. 25752680 10.1111/gcb.12916 PMC4676934

[nph70206-bib-0039] Franklin RS . 2013. Growth response of the alpine shrub, Linanthus pungens, to snowpack and temperature at a rock glacier site in the eastern Sierra Nevada of California, USA. Quaternary International 310: 20–33.

[nph70206-bib-0040] Gärtner H , Schweingruber FH . 2013. Microscopic preparation techniques for plant stem analysis (Originalausg). Remagen, Germany: Verlag Dr. Kessel.

[nph70206-bib-0041] Gauslaa Y . 1984. Heat Resistance and Energy Budget in Different Scandinavian Plants. Holarctic Ecology 7: 5–78.

[nph70206-bib-0042] German DA , Al‐Shehbaz IA . 2010. Nomenclatural novelties in miscellaneous Asian Brassicaceae (Cruciferae). Nordic Journal of Botany 28: 646–651.

[nph70206-bib-0043] Harris, IC . 2020: Cru Jra v2.1: a forcings dataset of gridded land surface blend of Climatic Research Unit (CRU) and Japanese reanalysis (JRA) data; Jan.1901–Dec.2019. Centre for Environmental Data Analysis.

[nph70206-bib-0044] Jevšenak J . 2020. New features in the dendroTools R package: bootstrapped and partial correlation coefficients for monthly and daily climate data. Dendrochronologia 63: 125753.

[nph70206-bib-0045] Jevšenak J , Levanič T . 2018. dendroTools: R package for studying linear and nonlinear responses between tree‐rings and daily environmental data. Dendrochronologia 48: 32–39.

[nph70206-bib-0046] Klimešová J , Doležal J , Dvorský M , De Bello F , Klimeš L . 2011. Clonal growth forms in Eastern Ladakh, Western Himalayas: classification and habitat Preferences. Folia Geobotanica 46: 191–217.

[nph70206-bib-0047] Körner C . 2017. A matter of tree longevity. Science 355: 130–131. 28082545 10.1126/science.aal2449

[nph70206-bib-0048] Körner C . 2021. Alpine plant life: Functional plant ecology of high mountain ecosystems, 3^rd^ edn. Cham, Switzerland: Springer.

[nph70206-bib-0049] Körner C . 2023. Concepts in alpine plant ecology. Plants 12: 2666. 37514280 10.3390/plants12142666 PMC10386573

[nph70206-bib-0050] Körner C , Riedl S , Keplinger T , Richter A , Wiesenbauer J , Schweingruber F , Hiltbrunner E . 2019. Life at 0°C: the biology of the alpine snowbed plant *Soldanella pusilla* . Alpine Botany 129: 63–80.

[nph70206-bib-0051] Krab EJ , Roennefarth J , Becher M , Blume‐Werry G , Keuper F , Klaminder J , Kreyling J , Makoto K , Milbau A , Dorrepaal E . 2018. Winter warming effects on tundra shrub performance are species‐specific and dependent on spring conditions. Journal of Ecology 106: 599–612.

[nph70206-bib-0052] Lawlor JA , Comte L , Grenouillet G , Lenoir J , Baecher JA , Bandara RMWJ , Bertrand R , Chen I‐C , Diamond SE , Lancaster LT *et al*. 2024. Mechanisms, detection and impacts of species redistributions under climate change. Nature Reviews Earth and Environment 5: 351–368.

[nph70206-bib-0053] Leng R , Harrison S , Anderson K . 2023. Himalayan alpine ecohydrology: an urgent scientific concern in a changing climate. Ambio 52: 390–410. 36324019 10.1007/s13280-022-01792-2 PMC9755440

[nph70206-bib-0054] McCarthy DP . 1992. Dating with cushion plants: establishment of a silene acaulis growth curve in the Canadian Rockies. Arctic and Alpine Research 24: 50.

[nph70206-bib-0055] Miehe G , Miehe S , Kaiser K , Reudenbach C , Behrendes L , Wang Y . 2019. Vegetation patterns and processes in the Western Tibetan Plateau and their relationship with environmental conditions. Journal of Biogeography 46: 987–1003.

[nph70206-bib-0056] Milla R , Giménez‐Benavides L , Escudero A , Reich PB . 2009. Intra‐ and interspecific performance in growth and reproduction increase with altitude: a case study with two Saxifraga species from northern Spain. Functional Ecology 23: 111–118.

[nph70206-bib-0057] Molenda O , Reid A , Lortie CJ . 2012. The Alpine Cushion Plant *Silene acaulis* as foundation species: a bug's‐eye view to facilitation and microclimate. PLoS ONE 7: e37223. 22655035 10.1371/journal.pone.0037223 PMC3360034

[nph70206-bib-0058] Myers‐Smith IH , Hik DS . 2018. Climate warming as a driver of tundra shrubline advance. Journal of Ecology 106: 547–560.

[nph70206-bib-0059] Pant GB , Kumar PP , Revadekar JV , Singh N . 2018. Climate change in the Himalayas, hardcover. Springer International Publishing AG. doi: 10.1007/978-3-319-61654-4. isbn:978‐3‐319‐61653‐7.

[nph70206-bib-0060] Pepin NC , Arnone E , Gobiet A , Haslinger K , Kotlarski S , Notarnicola C , Palazzi E , Seibert P , Serafin S , Schöner W *et al*. 2022. Climate changes and their elevational patterns in the mountains of the world. Reviews of Geophysics 60: e2020RG000730.

[nph70206-bib-0061] R Core Team . 2023. R: a language and environment for statistical computing (R v.4.2. 3). Vienna, Austria: R Foundation for Statistical Computing.

[nph70206-bib-0062] Rai S , Breme N , Jandova V , Lanta V , Altman J , Ruka AT , Rixen C , Dolezal J . 2024. Growth dynamics and climate sensitivities in alpine cushion plants: insights from Silene acaulis in the Swiss Alps. Alpine Botany. doi: 10.1007/s00035-024-00318-8.

[nph70206-bib-0063] Řeháková K , Čapková K , Dvorský M , Kopecký M , Altman J , Šmilauer P , Doležal J . 2017. Interactions between soil phototrophs and vascular plants in Himalayan cold deserts. Soil Biology and Biochemistry 115: 568–578.

[nph70206-bib-0064] Richards FJ . 1959. A flexible growth function for empirical use. Journal of Experimental Botany 10: 290–301.

[nph70206-bib-0065] Rixen C , Schwoerer C , Wipf S . 2010. Winter climate change at different temporal scales in Vaccinium myrtillus, an Arctic and alpine dwarf shrub. Polar Research 29: 85–94.

[nph70206-bib-0066] Rosbakh S , Poschlod P . 2018. Killing me slowly: harsh environment extends plant maximum life span. Basic and Applied Ecology 28: 17–26.

[nph70206-bib-0067] Ruka AT , Čapková K , Řeháková K , Angel R , Chroňáková A , Kopecký M , Macek M , Dvorský M , Doležal J . 2023. Bacterial and plant community successional pathways in glacier forefields of the Western Himalaya. European Journal of Soil Biology 119: 103565.

[nph70206-bib-0068] Schmidt S , Nüsser M . 2017. Changes of high altitude glaciers in the Trans‐Himalaya of Ladakh over the past five decades (1969–2016). Geosciences 7: 27.

[nph70206-bib-0069] Schmidt S , Nüsser M . 2023. Glaciers of Central Ladakh: distribution, changes and relevance in the Indian Trans‐Himalaya. In: Humbert‐Droz B , Dame J , Morup T , eds. Environmental change and development in Ladakh, Indian Trans‐Himalaya. Cham, Switzerland: Springer Nature Switzerland, 11–30. doi: 10.1007/978-3-031-42494-6_2.

[nph70206-bib-0070] Schmidt SK , Lipson DA . 2004. Microbial growth under the snow: implications for nutrient and allelochemical availability in temperate soils. Plant and Soil 259: 1–7.

[nph70206-bib-0071] Schweingruber FH , Dvorský M , Börner A , Doležal J . 2020. Atlas of stem anatomy of Arctic and Alpine plants around the globe. Cham, Switzerland: Springer.

[nph70206-bib-0072] Seneviratne SI , Zhang X , Adnan M , Badi W , Dereczynski C , Di Luca A , Ghosh S , Iskander I , Kossin J , Lewis S *et al*. 2021. Weather and climate extreme events in a changing climate (chapter 11). In: Masson‐Delmotte V , Zhai P , Pirani A , Connors SL , Péan C , Berger S , Caud N , Chen Y , Goldfarb L , Gomis MI *et al*., eds. IPCC 2021: climate change 2021: the physical science basis. Contribution of working group I to the sixth assessment report of the intergovernmental panel on climate change. Cambridge, UK and New York, NY: Cambridge University Press, 1513–1766.

[nph70206-bib-0073] Silvertown JW . 2013. The long and the short of it: The science of life span and aging. Chicago, IL: The University of Chicago Press.

[nph70206-bib-0074] Steinbauer MJ , Grytnes J‐A , Jurasinski G , Kulonen A , Lenoir J , Pauli H , Rixen C , Winkler M , Bardy‐Durchhalter M , Barni E *et al*. 2018. Accelerated increase in plant species richness on mountain summits is linked to warming. Nature 556: 231–234. 29618821 10.1038/s41586-018-0005-6

[nph70206-bib-0075] Stöcklin J , Bäumler E . 1996. Seed rain, seedling establishment and clonal growth strategies on a glacier foreland. Journal of Vegetation Science 7: 45–56.

[nph70206-bib-0076] Thakur D , Altman J , Jandová V , Fibich P , Münzbergová Z , Doležal J . 2024. Global warming alters Himalayan alpine shrub growth dynamics and climate sensitivity. Science of the Total Environment 916: 170252. 38253093 10.1016/j.scitotenv.2024.170252

[nph70206-bib-0077] Thayyen RJ , Dimri AP , Kumar P , Agnihotri G . 2013. Study of cloudburst and flash floods around Leh, India, during August 4–6, 2010. Natural Hazards 65: 2175–2204.

[nph70206-bib-0078] Von Arx G , Crivellaro A , Prendin AL , Čufar K , Carrer M . 2016. Quantitative wood anatomy—practical guidelines. Frontiers in Plant Science 7: 781. doi: 10.3389/fpls.2016.00781. 27375641 PMC4891576

