# Supplementary material for: Climate warming promotes growth in Himalayan alpine cushion plants but threatens survival through increased extreme snowfall
Source: New Phytol. 2025 May 12;247(1):115–27. doi: 10.1111/nph.70206 (PMC12138181; doi:10.1111/nph.70206)
Supplement: Supplementary file 1 — Fig. S1 Monthly average temperature trends from 1975 to 2022 at 5900 m elevation in the Himalayan subnival zone. Fig. S2 Century‐scale monthly temperature trends from 1900 to 2022. Fig. S3 Monthly precipitation trends from 1975 to 2022. Fig. S4 Long‐term trends in monthly precipitation sums from 1900 to 2022. Fig. S5 Correlation between in situ and CRU gridded daily temperatures. Fig. S6 Interannual near‐ground temperature variations (2009–2021) at 5900 m. Fig. S7 Interannual soil temperature dynamics (2014–2021) at 8 cm depth. Fig. S8 Ontogenetic growth changes in warmer and colder climates. Fig. S9 Correlations between growth and temperature and precipitation. Fig. S10 Correlations between recruitment and temperature and precipitation. Please note: Wiley is not responsible for the content or functionality of any Supporting Information supplied by the authors. Any queries (other than missing material) should be directed to the New Phytologist Central Office. [file NPH-247-115-s001.docx]

New Phytologist Supporting Information
Article title: **Climate Warming Promotes Growth in Himalayan Alpine Cushion Plants but Threatens Survival Through Increased Extreme Snowfall**

Authors: Veronika Jandova, Jan Altman, Hana Sehadova, Martin Macek, Pavel Fibich, Adam Taylor Ruka, Jiri Dolezal
Article acceptance date: 18 April 2025


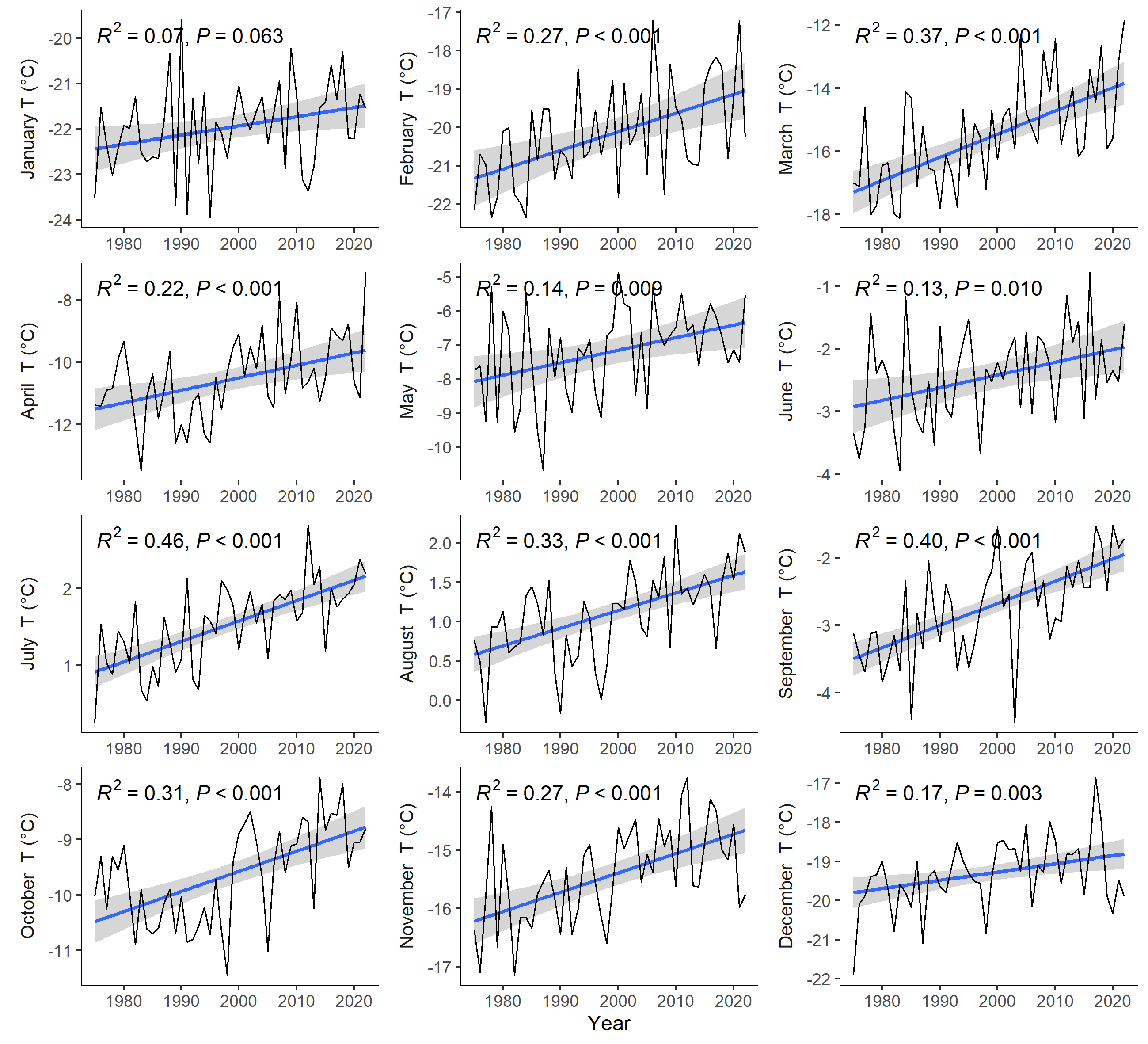


**Supporting Information Fig. S1.** The figure presents annual changes in monthly average temperatures over the past four decades (1975-2022), with each subplot representing a different month. The y-axis denotes temperature (T, in °C), while the x-axis represents the years. The black line illustrates observed temperature fluctuations over time, and the blue regression line, accompanied by a shaded confidence interval, indicates the overall warming trend. All months exhibit an increasing temperature trend, confirming a persistent warming pattern. However, the strength of this trend varies across months, as reflected in the coefficient of determination (R²) and p-values. The most pronounced warming trends are observed in July (R² = 0.46, P < 0.001), March (R² = 0.37, P < 0.001), and September (R² = 0.40, P < 0.001), indicating a strong correlation between time and rising temperatures. In contrast, January shows the weakest trend (R² = 0.07, P = 0.063), which is not statistically significant. The remaining months display statistically significant warming (P < 0.05), with moderate to strong R² values. Overall, the figure provides compelling evidence of significant warming trends throughout the year, particularly in the summer and transitional months, reinforcing the impact of climate change on temperature increases across all seasons.


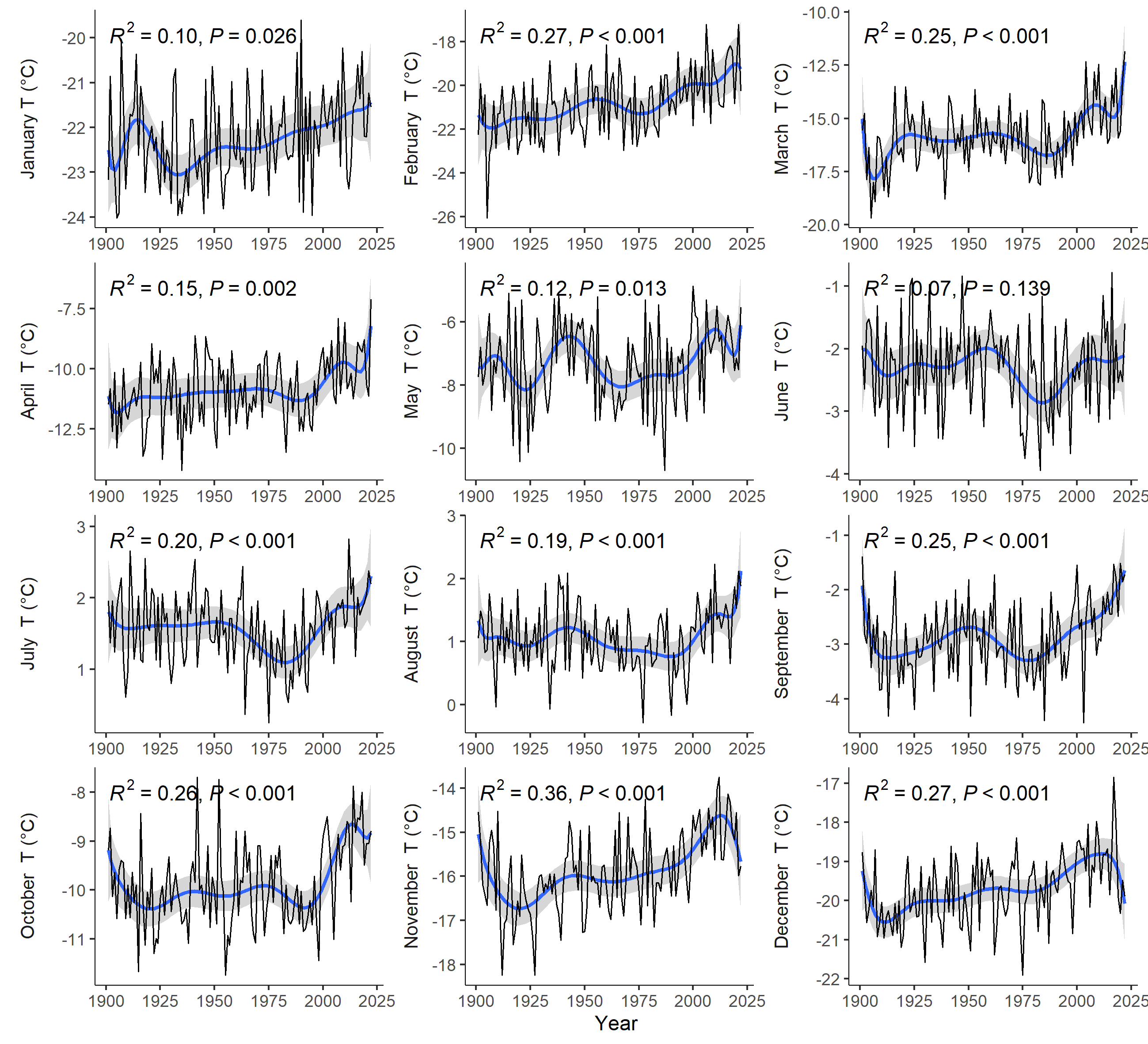


**Supporting Information Fig. S2.** The figure illustrates long-term trends in monthly average temperatures from 1900 to 2022, with each subplot representing a different month. The y-axis denotes temperature (T, in °C), while the x-axis represents the years. The black line indicates observed temperature fluctuations over time, juxtaposed with a 10-yr low-pass filtered representation (thick blue line), accompanied by a shaded confidence interval, to highlight broader climatic trends. Most months exhibit a warming trend, but the strength of these trends varies. The most significant warming is observed in November (R² = 0.36, P < 0.001), followed by March, September, October, and December (R² = 0.25–0.27, P < 0.001), indicating strong positive associations with time. Conversely, June shows the weakest trend (R² = 0.07, P = 0.139), which is not statistically significant, suggesting that temperatures in June have not increased consistently over the studied period. Other months, including January, April, May, and July, display statistically significant but weaker warming trends (R² = 0.10–0.20, P < 0.05). Overall, the results indicate a general warming trend across most months, with the most substantial increases occurring in autumn and early winter.


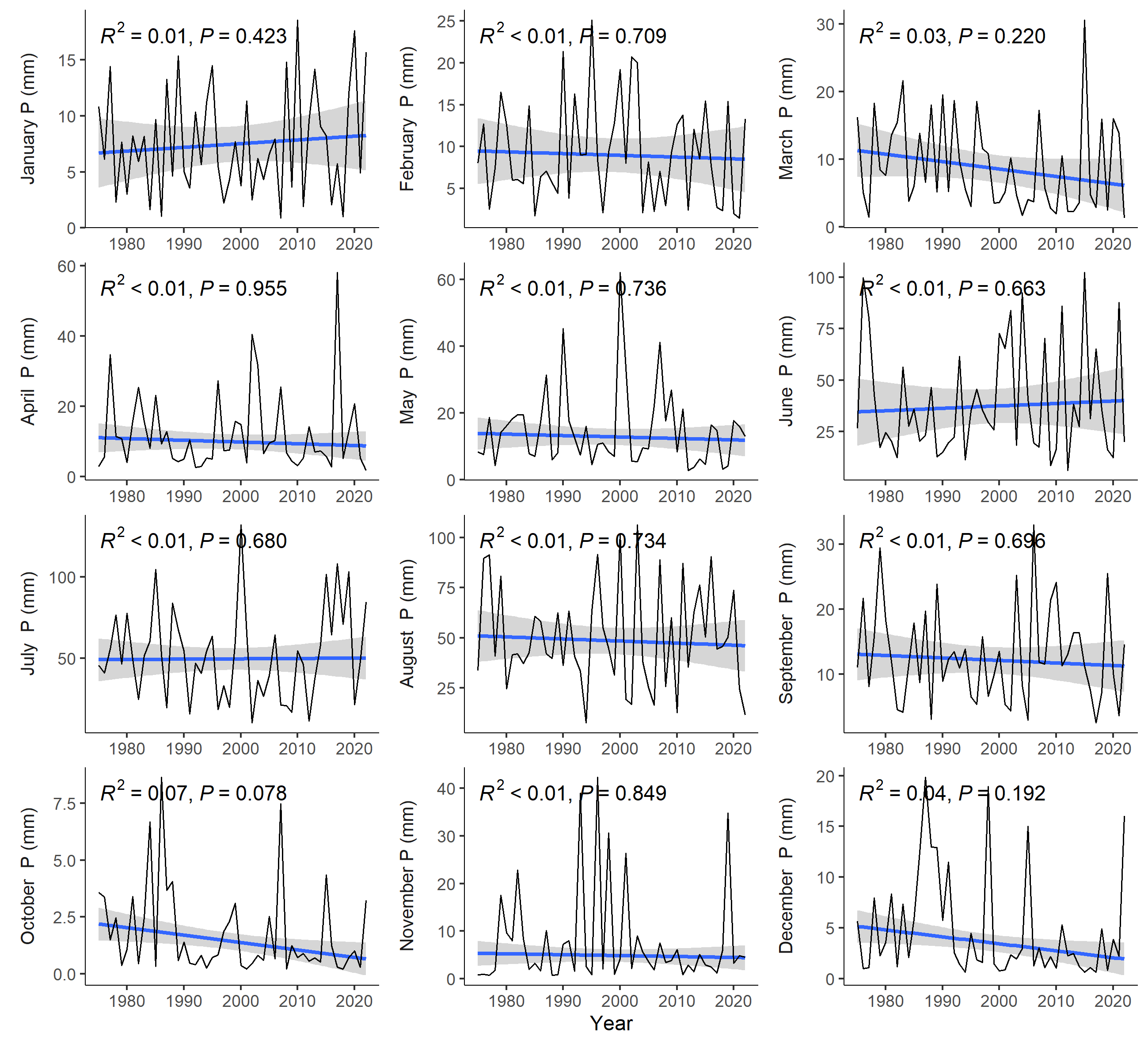
**Supporting Information Fig. S3.** The figures depict the temporal trends in monthly precipitation (P) from 1975 to 2022. The x-axis represents the years, while the y-axis indicates precipitation in millimeters. Each subplot corresponds to a specific month, arranged from January to December. The black lines in each panel illustrate the observed precipitation data over time, while the blue lines represent regression lines, with gray-shaded areas indicating confidence intervals.


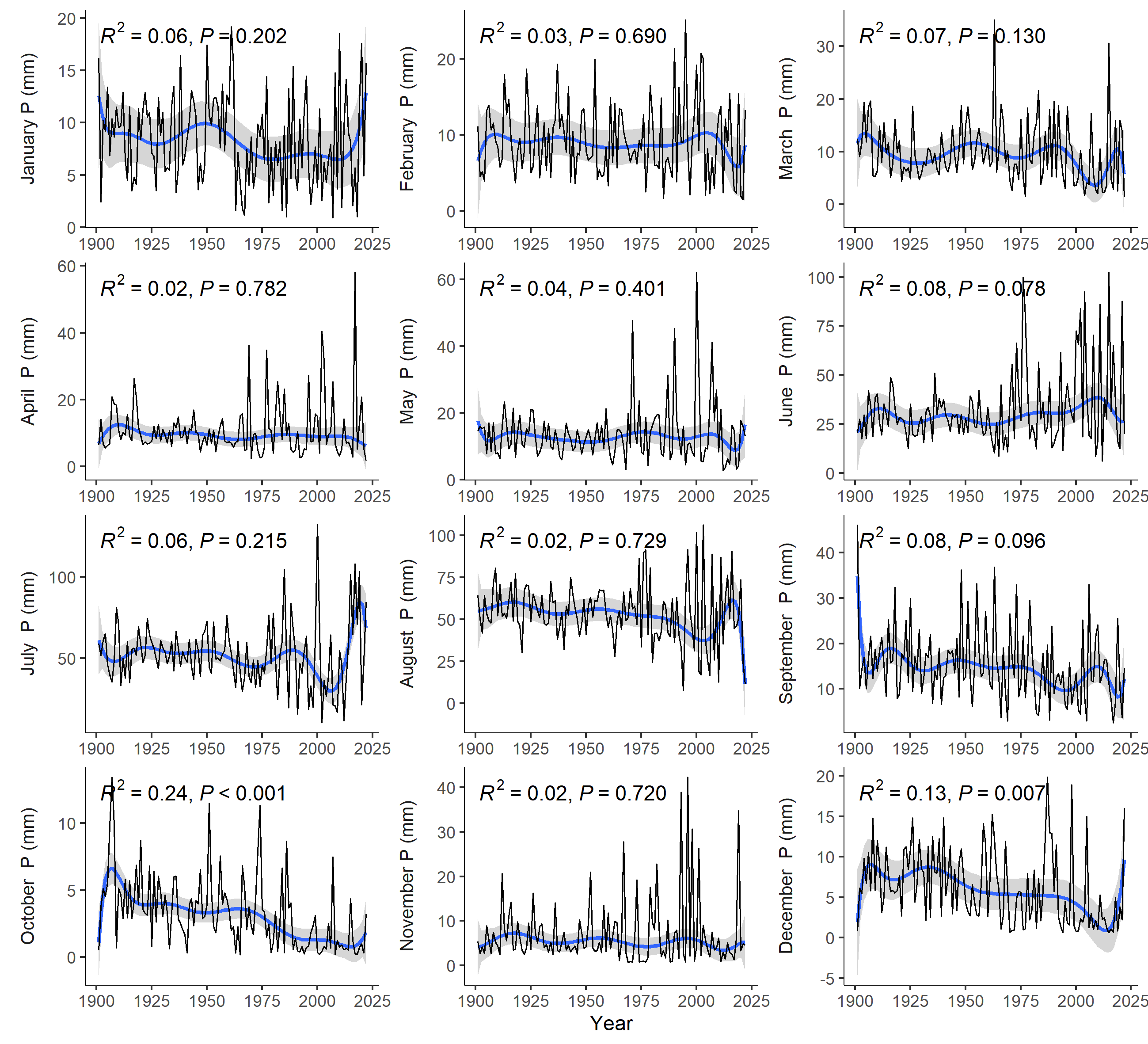


**Supporting Information Fig. S4.** The figure illustrates long-term trends in monthly precipitation sum from 1900 to 2022, with each subplot representing a different month. The y-axis denotes precipitation (P, in mm), while the x-axis represents the years. The black line indicates observed precipitation fluctuations over time, juxtaposed with a 10-yr low-pass filtered representation (thick blue line), accompanied by a shaded confidence interval, to highlight broader climatic trends.


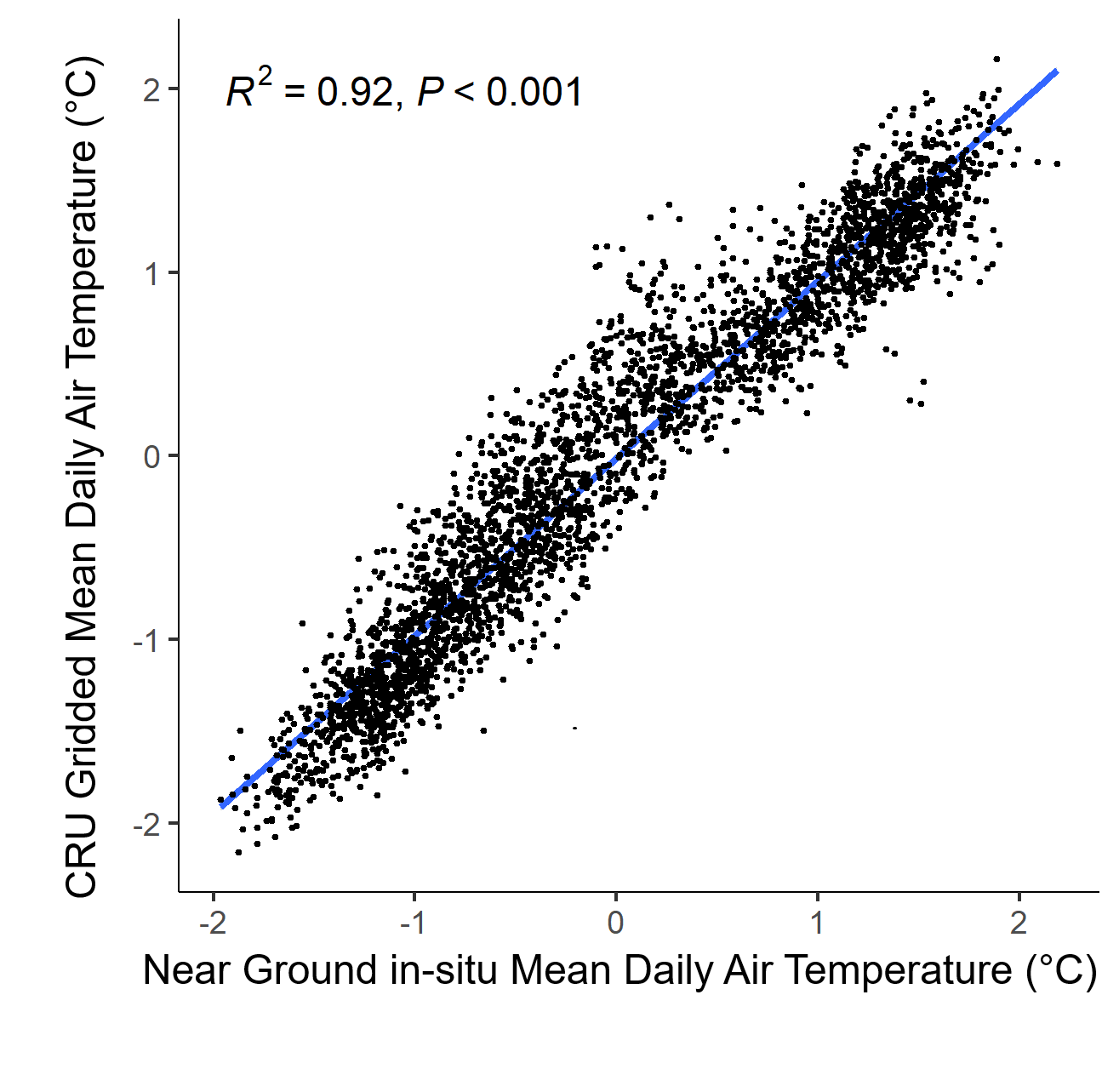


**Supporting Information Fig. S5.** A scatter plot comparing near-ground in situ mean daily air temperature (x-axis) with CRU gridded mean daily air temperature (y-axis). Each black dot represents a daily temperature measurement, and the blue regression line illustrates the strong linear correlation between the two datasets (R² = 0.92, P < 0.001). The high coefficient of determination (R²) indicates a strong agreement between in situ temperature recordings and the CRU gridded dataset, confirming the reliability of using gridded climate data to analyze long-term temperature trends in the study region.


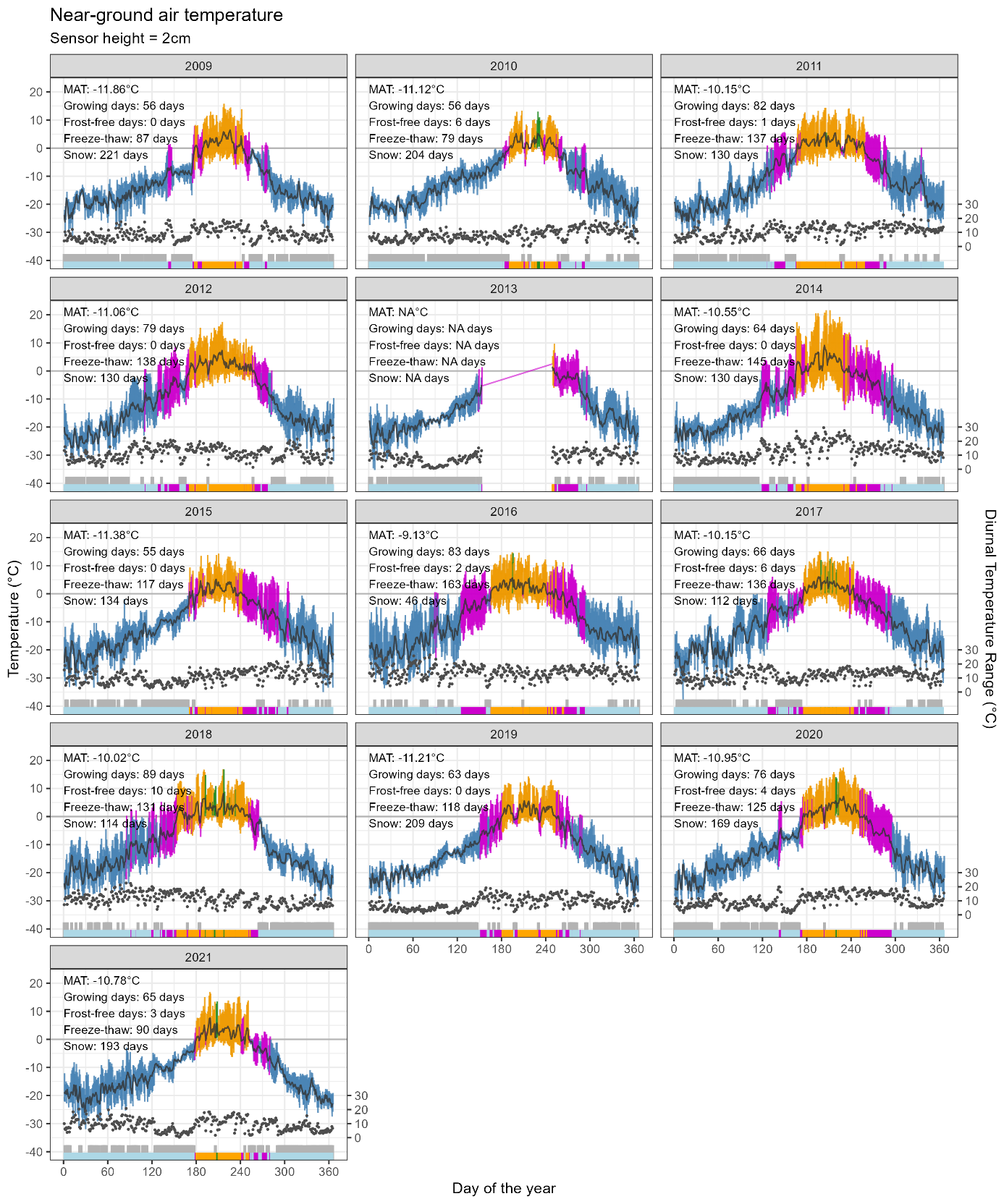


**Supporting Information Fig. S6.** Near-ground air temperature variations at 5900 m in eastern Ladakh above Tso Moriri Lake, at a height of 2 cm above the ground, recorded using Onset HOBO U23 Pro v2 in different year (2009–2021). The x-axis represents the day of the year, while the y-axis shows temperature in degrees Celsius (°C). Each panel contains a color-coded time series of temperature data: blue represents the ice days, green represents frost-free days (when the average daily temperature remains above freezing), orange represents freeze-thaw days with the average daily temperature above freezing, while red represents freeze-thaw days when the mean daily temperature remains below 0°C. Black dots indicate daily temperature ranges. Summary statistics in the top left corner include MAT (Mean Annual Temperature), Growing days (conditions suitable for plant growth), Frost-free days, and Freeze-thaw days. A bottom strip highlighting snow cover duration is based on a range < 12°C and Tmax < 1.5°C ( in shade), which aligns quite well with the snow estimated from RH (RH > 70%). From the data, variations in temperature dynamics across different years can be observed. In colder years, such as 2009 and 2019, there were more days with snow cover (221 and 209 days, respectively) and fewer growing days. Conversely, in warmer years, such as 2016 and 2018, there were more growing days (83 and 89 days, respectively) and fewer snow days. The year 2013 lacks complete data, indicated by "NA" values for key metrics. A notable trend is the variability in the number of freeze-thaw days, which fluctuates across years, ranging from 68 days (2015) to as high as 163 days (2016). Similarly, snow cover duration varies significantly, with years like 2016 having only 46 snow days, while 2009, 2010 and 2019 had prolonged snow cover exceeding 200 days.


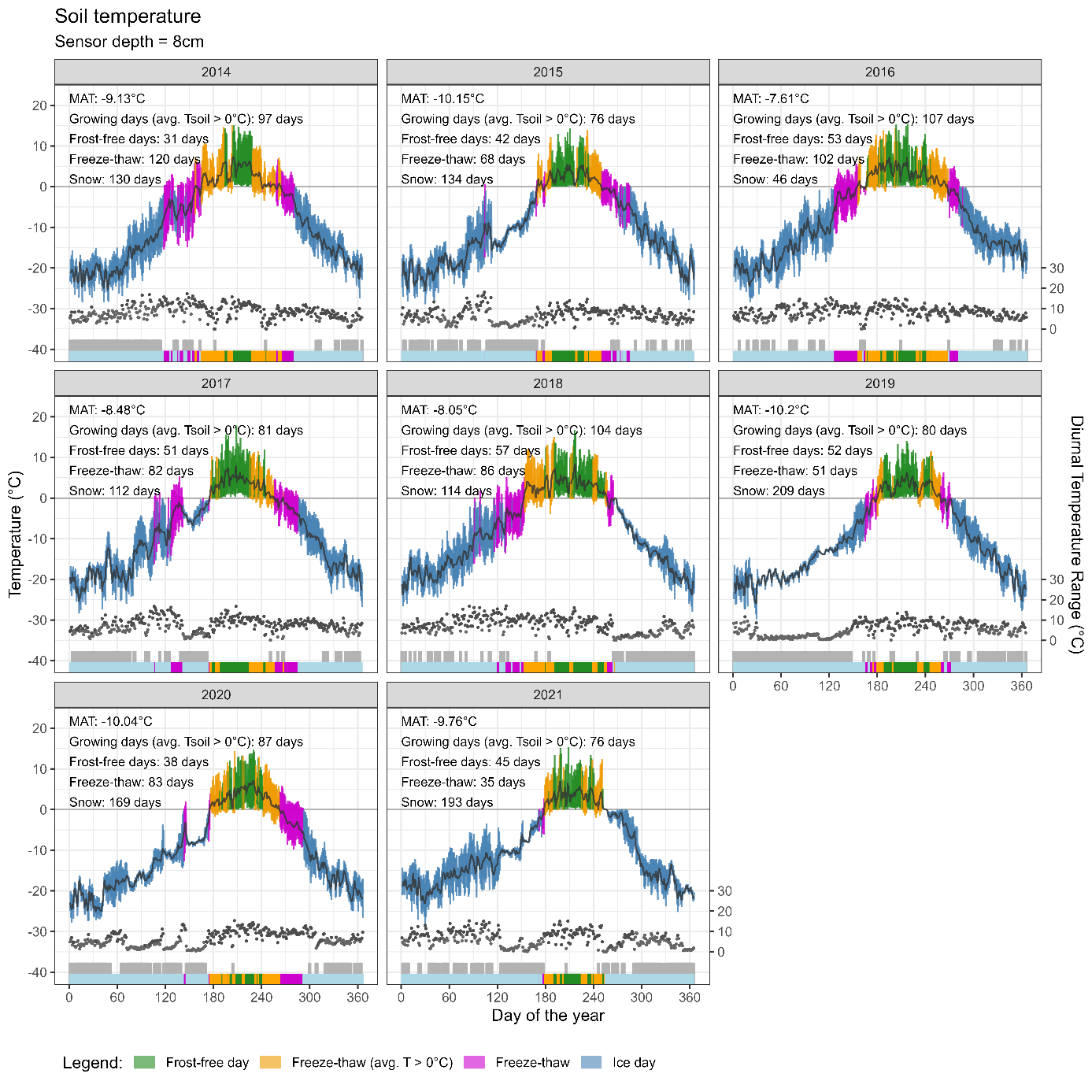


**Supporting Information Fig. S7.** Soil temperature variations at a depth of 8 cm across multiple years from 2014 to 2021, measured using Onset HOBO U23 Pro v2 climate data, corresponding with rooting zone of studied *Ladakiella* *klimesii* plants. Each panel represents a different year and includes temperature trends, growing season characteristics, and freeze-thaw dynamics. The number of growing days, defined as periods when the soil temperature exceeds 0°C, fluctuates significantly, with the highest recorded in 2016 (107 days) and 2018 (104 days), whereas 2015 (76 days) and 2021 (76 days) exhibit shorter growing seasons.

The number of frost-free days, where temperatures remain above freezing, also shows notable variation, ranging from as few as 31 days in 2014 to as many as 57 days in 2018. The duration of freeze-thaw cycles, characterized by alternating freezing and thawing conditions, is longest in 2014 (120 days) and shortest in 2021 (35 days), indicating interannual variability in soil thermal regimes. Snow cover duration also fluctuates, with years like 2019 (209 days) and 2021 (193 days) experiencing prolonged snow periods, whereas 2016 (48 days) exhibit much shorter snow-covered periods. The temperature trends across all years exhibit a seasonal pattern, with clear warming during the middle of the year and cooling toward the beginning and end. The diurnal temperature range, shown on the right y-axis, displays significant fluctuations, particularly during transitional periods. The color-coded legend at the bottom distinguishes different thermal phases, with green indicating frost-free days, yellow and red denoting different freeze-thaw conditions, and blue representing ice days. Overall, the figure illustrates the interannual variability in soil temperature dynamics, growing season length, and snow cover, reflecting the impacts of climate variability on subsurface thermal conditions.


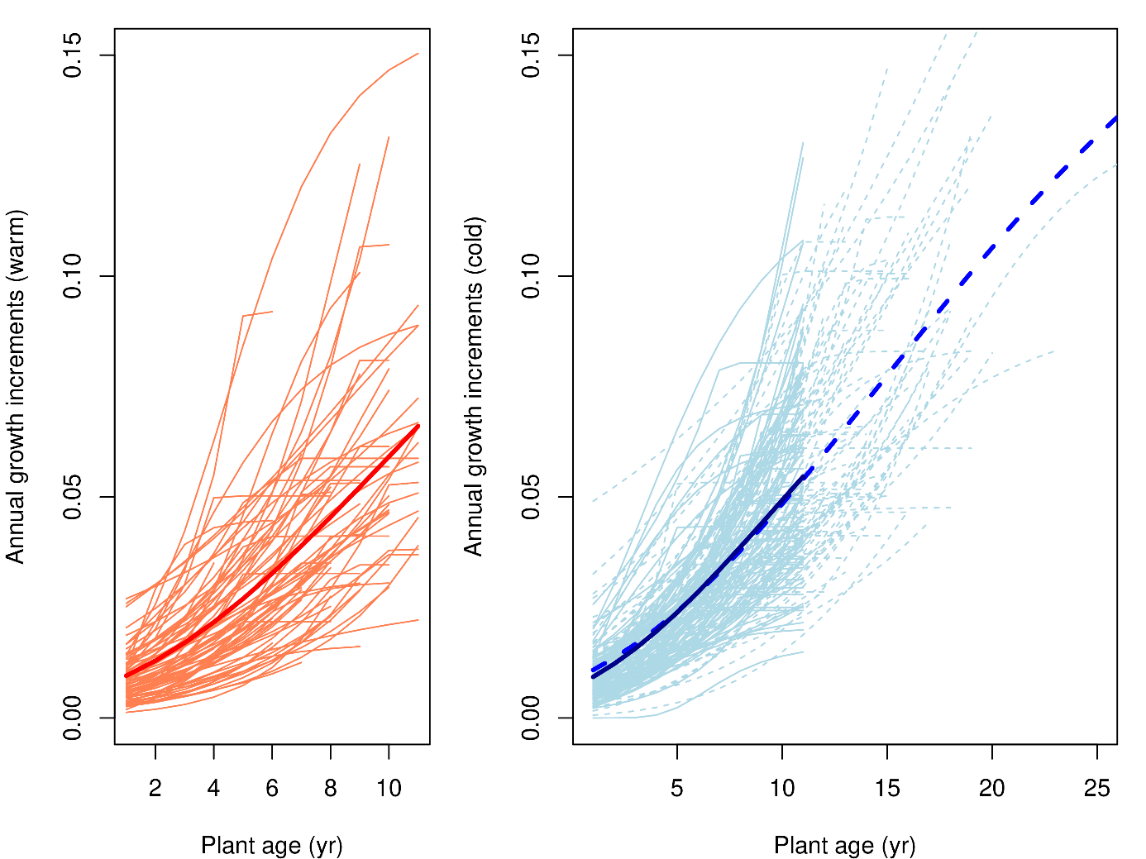


**Supporting Information Fig. S8.** The figure compares annual growth increments against plant age under two climate conditions: warmer (left panel, red) and colder (right panel, blue). In warmer climates, plants exhibit rapid early growth, with increments increasing steeply and reaching a maximum age of around 10 years. Individual growth trajectories vary, but the overall trend suggests accelerated development. In contrast, plants in colder climates grow more gradually over a longer lifespan, with growth increments rising steadily beyond 20 years. The greater variation in individual growth patterns indicates a more prolonged growth phase. These results highlight a growth-longevity trade-off, where faster early growth in warmer conditions may come at the cost of reduced lifespan, while slower growth in colder environments enhances long-term persistence. The findings align with climate-driven shifts in plant growth strategies, suggesting that warming accelerates early development but may ultimately limit plant longevity and maximum size.


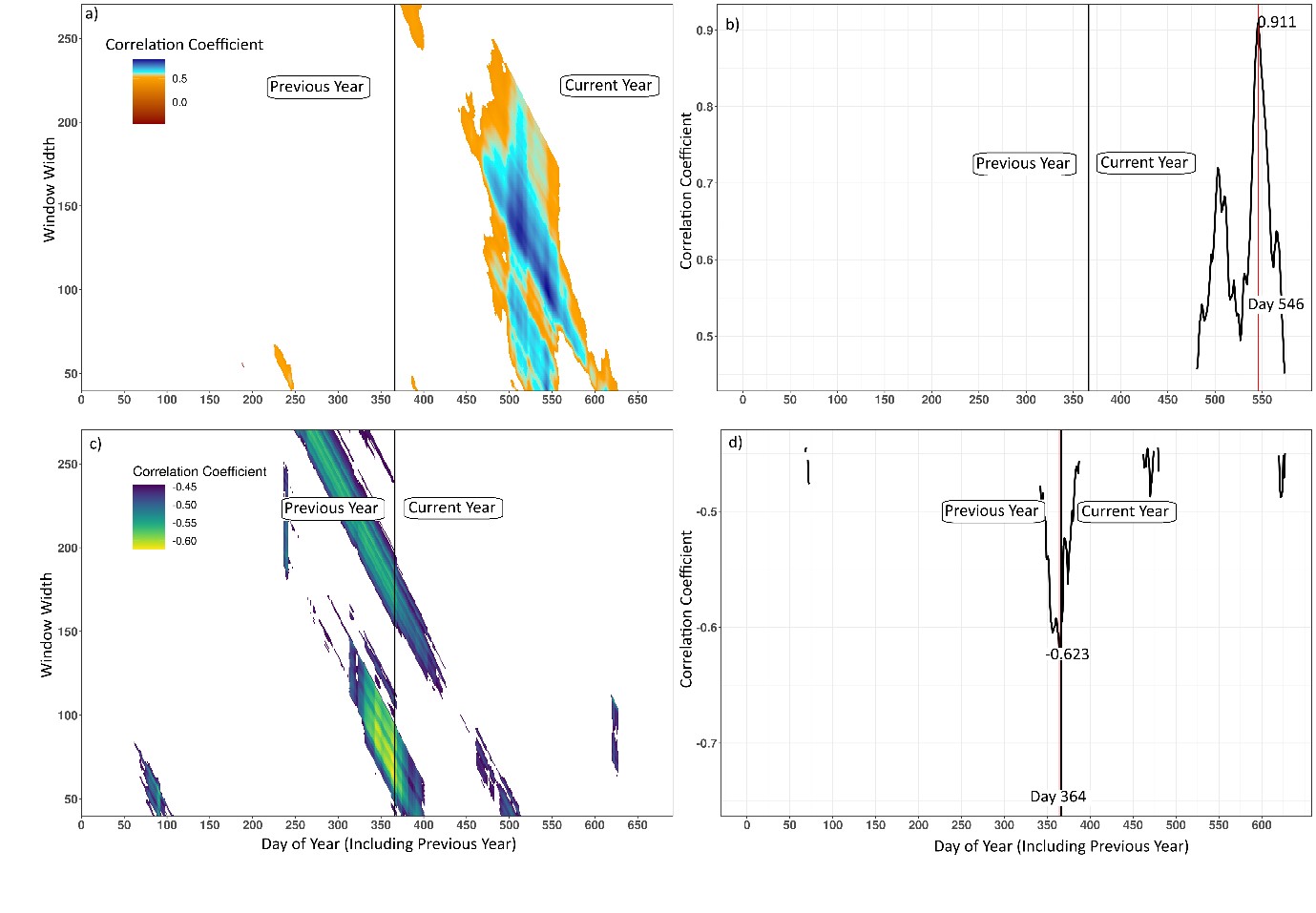


**Supporting Information Fig. S9.** Heatmaps showing the correlation between growth and (a) temperature and (c) precipitation (1989-2017). The running optimal window width detecting the strongest correlations is shown, with temperatures between June 29 and October 4 (b) and precipitation between December 30 of the previous year and March 11 (d) being the most influential.


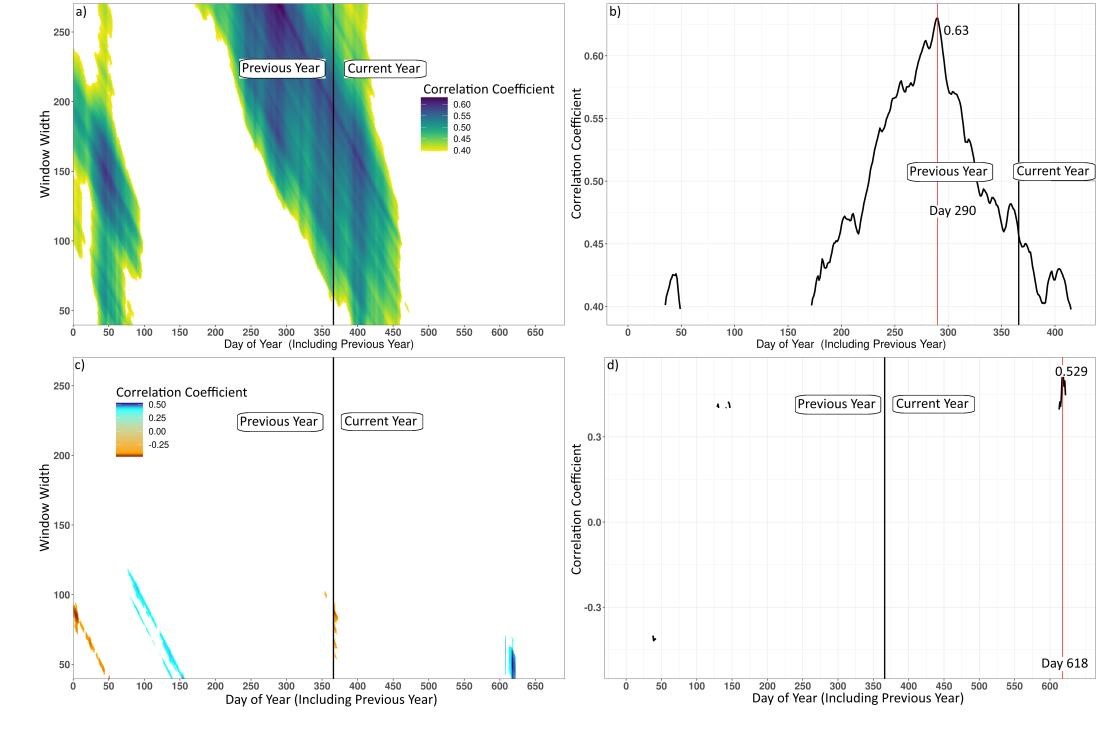


**Supporting Information Fig. S10.** Heatmaps showing the correlation between recruitment and (a) temperature and (c) precipitation over 29 years (1989-2017). The running optimal window width detecting the strongest correlations is shown, with temperatures between October 17 of the previous year and July 12 (b) and precipitation between September 9 and October 27 (d) being the most influential.
